# Abstracts of papers presented at the ISLAR (International symposium on laboratory automation and robotics) 1997

**DOI:** 10.1155/S1463924698000066

**Published:** 1998


Journal of Automatic Chemistry, Vol. 20, No. 2 (March-April 1998) pp. 31-66

### Abstracts of papers presented at the ISLAR (International Symposium on Laboratory Automation and Robotics) 1997

The 15th International Symposium on Laboratory Automation and Robotics was held from 19–22 October 1997 in Boston, USA. State-of-the-art developments in laboratory automation and robotics were reflected in the symposium programme, which included papers and posters on all aspects of the technology—drug discovery research, data handling and data management, chemical analysis, re-engineering the laboratory, laboratory workstations, bioanalytical assays, managing laboratory automation, dissolution testing, pharmaceutical analysis, automation and combinatorial chemistry, validating automated methods and advanced topics. We are printing abstracts of papers from ISLAR and we hope that you find them informative and productive.

The 1998 ISLAR will be held in Boston, USA from 18–21 October 1998. Session topics will be similar to previous years, but will also include high throughput screening, re-engineering the laboratory, and increasing productivity. For more information, contact Christine O'Neil at 508 497 2224; fax on 508 435 3439; send an e-mail to islar@ISLAR.com or visit the ISLAR pages at http://www.islar.com.

### Technology and the new high-throughput drug discovery paradigms

*Andrew Shaw, Zeneca Pharmaceuticals, Wilmington, DE, USA*

The development of powerful new research technologies has led to greatly enhanced expectations of the discovery phase of pharmaceutical research. However, as well as bringing the promise of new efficiency, the rapid pace of new technology development and implementation constantly creates new bottlenecks in the research process, threatens competitive obsolescence in the technology platform and creates questions about organizational structure. This presentation gave a personal overview of the problems of creating and maintaining an effective capability in the lead discovery phase of research, while highlighting areas for technology development.

### Potential applications for laboratory automation in drug discovery and development: a drug metabolism perspective

*Gerald T. Miwa, Dupont Merck Pharmaceutical, Newark, DE, USA*

Analytical throughput capacity is a common limiting resource in conducting pharmacokinetic studies. Pharmacokinetic studies are an integral part of non-clinical toxicology studies, as well as clinical pharmacology research. For many pharmaceutical companies, pharmacokinetic studies are also becoming an integral part of the drug discovery paradigm. In contrast to drug development, where throughput requirements are dictated by many samples from few compounds, drug discovery is characterized by few samples from many compounds. This difference affords new applications for automation during drug discovery. The future direction for these applications at DuPont Merck was described.

### Success is not necessarily automatic

*Alastair Selkirk, Abbott Laboratories, North Chicago, IL, USA*

The pharmaceutical industry needs to not only discover new molecules but also to develop them as efficiently and effectively as possible. Success is being first to market, not necessarily first to proof of concept. To be successful, development needs to reduce timelines while maintaining quality and preventing burnout. While automation obviously plays a very significant role in this approach, many other factors must be in place for automation to be as effective as possible. These factors include planning, process optimization, organizational structure, people development and the need to see the total picture.

This presentation discussed these factors and their relationship with automation. It evaluated less obvious areas of automation, like document management, as well as the more established ones and discussed the premise, that it is the integration of all these aspects, including automation, that truly offers the biggest opportunities.

### Re-inventing drug discovery: issues and actions in the quest for innovation and productivity

*Pradip Banerjee, Andersen Consulting, Florham Park, NJ, USA*

As it approaches the 21st century, the pharmaceutical industry has re-emphasized its focus on innovative drug discovery as the key to future success. Prioritizing innovation has become a strategic imperative in an increasingly competitive environment, and the hurdles for success are extremely high.

In order to sustain revenue growths at the 10% level that the industry has historically produced, the major global pharmaceutical players must average between 5–6 significant (that is, with a sales potential of greater than \$350 M/year) NCE launches annually in the future. Andersen Consulting has conducted a study of how pharmaceutical companies must restructure their drug discovery programmes to develop the high performance drug discovery processes necessary for future success. The

0142-0453/98 \$12·00 © 1998 Taylor & Francis Ltd objectives of the study were to understand the future direction of the discovery research process, analysing the impact of new technology, the implications for process organization and management, and the goals that cutting-edge discovery companies are setting themselves for the future. A broad survey was undertaken of discovery companies, with a representative sample of ten companies ranging from the top-tier pharmaceutical majors, through middle and lower tier players to biopharmaceutical companies, in the US, Europe, and Japan. One hundred interviewees covered all aspects of involvement with the discovery process, among them senior management of the R&D function and therapeutic areas, and individuals in biological, chemical, development and IT functions.

The Andersen Consulting study highlights the intimidating scale of the task facing discovery companies. In order to maintain the goals set to maintain market competitiveness, more NCE candidates for clinical development must be produced, and faster. New goals set by companies surveyed are a minimum of one NCE per 100 discovery research staff, and a doubling of the speed of delivery of NCEs to clinical development, from up to seven years to less than three. Quality must not be compromised by quantity; company goals are more development-ready compounds to lower the attrition rate of clinical development, and, ultimately, to ensure that the launch of truly innovative products to reap maximum reward on investment.

The effective exploitation of the new technologies transforming the drug discovery process will be a key to meeting these goals. It is clear from Andersen Consulting research that the key to high performance drug discovery is as much about the development of effective processes, information management, and people organization as it is about new technologies. Andersen Consulting has developed recommendations for an approach to high performance drug discovery which optimizes the interaction of these elements.

Effective co-ordination of technology, process and people will require an IT integration strategy which optimizes the potential of all three. Knowledge, rather than information management must be the key goal, with comprehensive and integrated applications to support the whole process from target generation and characterization, lead identification and optimization, and the discovery/development interface.

### Placing and preserving priorities: projects, productivity, progress and people

*John Babiak, Wyeth-Ayerst Research, Princeton, NJ, USA*

Robotic High Throughput Screening (HTS) within pharmaceutical companies has become a valuable resource to search for proprietary chemical structures which interact with novel biomolecular targets such as enzymes, cellular receptors or ion channels. Under some circumstances these chemicals may serve as leads which can be optimized into marketable drugs. To maximize success it is necessary to use HTS in a manner which complements and facilitates the changing priorities of drug discovery research.

Numerous forces compete for the limited resources of a HTS group. Major factors which can influence priorities are projects, productivity, progress and people. The challenge to the HTS group is to provide excellent and timely screening services, while continuing to devote efforts to new technologies and personnel development.

There are a great variety of issues considered by senior management in determining the priority of the project associated with a particular screen. Examples include the medical need for a treatment, anticipated market size for the expected drug, the status of related activities by competitors, validity of the molecular target as a mediator of the disease and perceived case of development. Since it is often the case that the projects with the greatest potential rewards have the lowest probability of success, it is not surprising that priorities among projects can change dramatically and quickly.

HTS groups are frequently evaluated by numerical measures of productivity, such as samples tested per week and number of screens completed per year. Naturally, this emphasis on throughput encourages the HTS group to prioritize in favour of screen designs which are easier to implement and run. Although in many cases conversion of assays into preferred screen formats will improve efficiency, some molecular targets may be deprioritized because they will decrease the perceived productivity of the HTS group.

Progress in developing new technologies and a commitment to development of the people within the HTS organization are two other factors which must also influence the setting of priorities. Evaluating and introducing new technologies—hardware, software, assay formats—is time consuming and reduces productivity in the short term. When successful, however, the results may be improved productivity or the ability to perform screens previously deprioritized for being too difficult.

People development must be a major concern, because it is the people who develop and implement the new technologies and determine how productive the robotics and automated equipment will actually be. HTS is most successful meeting the priorities for drug discovery research through the ability of the people in the HTS group to understand the needs of investigators on a variety of drug discovery projects, and the progress derived from the new technologies and to achieve the highest level of productivity possible.

### Planning and establishment of a high throughput screening site

*Julie J. Tomlinson, Brent T. Butler, Joan Frezza, Albert A. Smith, and W. Blaine Knight, Molecular Biochemistry, Glaxo Wellcome, Research Triangle Park, NC, USA*

During 1966 and 1997 Glaxo Wellcome US Research planned and implemented a new strategy. In the first quarter of 1996, 13 cross-functional work groups planned specific, individual components of the strategy and submitted reports detailing their plans to management. Management used the reports to devise an overall implementation plan, then Research was reorganized and implementation of the strategy commenced.

An important part of the strategy entailed development of two automated high throughput screening (HTS) sites in Research Triangle Park, NC. Both screening sites are a part of the Biochemistry division, one in a primary R&D complex and the other in a satellite building several miles away. This presentation described the set-up of the site in the satellite building.

Site set-up entailed hiring and training people, building a new laboratory and modifying existing labs, purchasing, installing and implementing automated systems and developing the process required to sustain compound handling with high throughput screening operations. All of these aspects of the screening site were developed and implemented simultaneously and successfully during 1996 and 1997.

### Making laboratory automation work for HTS

*Carol Ann Homon, Boehringer Ingelheim Pharmaceuticals, Inc., Ridgefield, CT, USA*

HTS today is quite different from what it was 10–12 years ago. Then, the goal was to screen a subset of the company's compound collection of 10 000 compounds or so. There was not a tremendous push to get these compounds tested, since not all scientists were firm believers in the HTS concept and not all programmes took advantage of HTS. Originally, an HTS laboratory could meet its throughput demands with a few simple automated pipetting machines such as the Tecan 500 series. These little machines turned out to be incredibly reliable workhorses. However, as HTS emerged as a successful new approach to new drug discovery, it became clear that more than a select few compounds needed to be screened. Companies started screening their entire collections ranging from 100 000 to 200 000 compounds or more. There also evolved a need to have screening results sooner so throughputs quickly rose. The higher throughputs required more automation, and in some cases, full robotic applications could be used to gain 16–24 hour a day operation. Problems for these systems came when they were required to do too many different types of assays. It became clear that each assay had its particular automation requirements. It was necessary to establish a system dedicated to each type of assay to provide a more robust and reliable system.

Even so, laboratory robotics had and still has problems. HTS assays were simplified and reduced to pipet and quantitate assays whenever possible. These homogeneous assays are highly dependent on the robotic pipetting device and on the quantitation device. The throughput is determined by how rapidly these steps are performed. Robotic pipettors have evolved to at least 8-tip instruments with 96-tip pipettors gaining a major foothold in HTS laboratories. The robotic pipettors are truly the heart of the HTS effort. In some cases, it has become necessary to have the pipettor built within the machine such as with FLIPR which is a fluorescent imaging system for whole cell assays. These specialized 'workstation' type devices need to be available within the HTS laboratory since a wide variety of cellular and molecular assays make up the assay profile in HTS.

The more recent push to generate even larger libraries through combinatorial chemistry has again set higher throughput demands. The future may well be UHTS which will require new robotic concepts to meet the demands for robustness of these systems. Clearly, the days of a single robot running up and down a single track will soon be done. The HTS laboratory must incorporate new technology and new automation as it becomes available to prepare for the demands of tomorrow as well as today. Managing this often rapid turnover of automation requires that HTS labs always be ready to adapt and change as needed.

### Available options for doing more with less: laboratory automation as one tool in the arsenal

*Stephen Scypinski, John Baiano and Theordore Sadlowski, Hoffmann-La Roche Inc., Nutley, NJ, USA*

As anyone involved in the pharmaceutical analysis aspect of drug development knows, projects that require analytical support can evolve from a number of different situations, some of which include:

- New molecular entities from drug discovery.
- Process changes.
- Packaging changes.
- Site changes.
- Line extensions.
- Unlicensed projects and compounds.

Laboratory automation has been shown to provide a viable and practical solution to assisting in analytical development. However, it is not always the most logical answer. A truly flexible and responsive analytical unit will make a decision on a case-by-case basis, when faced with a new project, whether it is best to:

- Automate some or all aspects/testing involved.
- Contract out to a reputable and approved Contract Research Organization (CRO).
- Hire temporary help.
- Use available in-house resources.
- Use a combination of the options above (for example, evaluate the complexity of the new project versus what the in-house resources are currently working on).

In this presentation, emphasis was placed on an evaluation of various situations and suggested options for the most effective use of resources. The role of automation as one of the important tools in the arsenal of these options was highlighted.

### Managing automation development in harmony with the rest of an international pharmaceutical company—A QC laboratory manager's perspective

*Paul Newton, Glaxo Wellcome Inc., Zebulon, NC, USA*

Transition from managing laboratory automation development of test methods for pharmaceutical products within the QC laboratory at one manufacturing site to participative management with other manufacturing and development groups offers significant opportunities and challenges. Areas to consider before taking on a company-wide automation development harmonization programme were offered. The approach that Glaxo Wellcome is taking for international harmonization of automation development for pharmaceutical analysis was presented. Specific examples of automation that are being affected by this co-operative effort, the experiences encountered up to this point, and potential benefits that will be delivered were discussed.

### Managing a robotics laboratory in a QC environment

*Iltifat Hasan, Matt Citardi and Muhammad Alburakeh, Barr Laboratories, Pomona, NY, USA*

Managing a laboratory is difficult enough. In order to fully benefit from the implementation of laboratory robotics, it is essential to understand robotics, and automation in general. While many managers understand (or think they do) the benefits of automation, they often do not realize the types of problems that robotic implementations can create. Automation provides varying challenges in validation, trouble-shooting, maintenance, training and regulatory compliance that cannot be overlooked. This presentation covered the management of robotic systems, including the up-front work necessary to properly integrate them into a QC laboratory environment.

### Laboratory automation—Some perspectives on the challenges in the implementation of the technology in pharmaceutical development

*Nigel North and Simon Smith, Smithkline Beecham Pharmaceuticals, Harlow, Essex, UK*

The intensifying pressure on reducing development time for new pharmaceutical products is resulting in an increasing need for laboratory automation. A key element for the successful implementation of robotics for product analysis is the establishment of a reliable process for interaction of the automation team with its various customers (for example, development product team and manufacturing group). Major Phase 2/3 products are targeted for robotics, these methods are used for stability studies to support regulatory filings. The reduction of cycle time for product development appears to be resulting in more stability studies to support NDA/MAA filings for several reasons. First, key clinical information may not be available before initiation of the stability studies and, second, simultaneous world-wide development may result in an increase in the number of product strength and pack options.

Technology transfer of robotic analytical methods to manufacturing is also more facile providing the manufacturing group has made the strategic decision to mirror the robotic equipment available in the development team. At present, few contract testing laboratories have robotic automation available which restricts outsourcing of large stability studies using this technology. Some perspectives on the challenges to maximizing the benefits of laboratory automation described above were discussed.

### Creative design and implementation of automated microbial susceptibility testing

*Chris Bierman and Theresa Kajs, Procter & Gamble, Mason, OH, USA*

Microbial susceptibility testing (MST) is a government mandated stability indicating analysis designed to test the efficacy of preservative systems with product matrices. The analysis requires that products packaged in MultiDose containers can be challenged with, at minimum, five USP specified microbes at a specific concentration. Products are subsequently plated over a 28-day period to check for microbial recovery and are deemed pass or fail based on the log reduction from the initial microbial concentration in product. The analysis can be broken down into three specific tasks:

- Sample preparation and innoculation.
- Sample plating.
- Data collection and results dissemination.

Health Care Microbiology at Procter & Gamble worked with Bohdan Automation to automate the sample plating procedure of MST as written in USP XXIII. This presentation discussed the design and validation of the two-robot system.

### Assessing climacteric ethylene of 100 million boxes of apples

*Eric Curry, USDA/Agriculture Research Service, Wenatchee, WA, USA*

The apple is a remarkable fruit. If harvested at optimum fruit storage potential, it is not uncommon to store them for at least 12 months under proper temperature and atmospheric conditions. Correct timing of harvest is predicated on defining the maturity of the apple, or stage of ripeness, which is determined by measuring fruit ethylene biosynthesis. Presently, the task of the apple industry is to assess the ripening status of about 10 billion apples in a six-week period to determine how to best store them for optimum fruit quality. First attempts to measure ethylene were done using bulk samples placed in a sealable container from which a headspace sample was taken periodically. This method was too qualitative, because one apple could be riper than the others, thereby generating most of the ethylene. The next attempt was to measure ethylene from individual fruit by taking a small sample of gas from the core with a large needle. This method reduced error; however, the number of samples grew prohibitively large. The method under design of assessing individual fruit is to place each apple in a small, 3-litre Plexiglas chamber with a low flow of scrubbed air. Microprocessors will allow the sampling of effluent gas as often as required 24 hours a day. This takes one person about 1 h to set up the system in order to accomplish the sampling of hundreds of apples that manually took about 2–3 min per sample. The greater number of samples both reduces error and increases the likelihood that apples will be in the consumer's hand with optimum quality.

### Modified automated titration system equipped with an ultrasonic homogenizer for the assay of absorbing gel materials in diapers

*Kyoko Ida, Shunji Ishigami, N. Nakagawa, Procter & Gamble Far East Inc., Kobe, Japan*

The current system for quantitative determination of Absorbing Gel Materials (AGM) in infants' diapers (nappies) utilizes air bubbling in the extraction procedure followed by acid–base titration. However, two issues have arisen. One is a corrosion problem on stainless steel frame due to acid vapour coming from the extraction bath. The other is an increased number of samples beyond the capacity of the current system. To overcome this situation, we modified the system. The bath was covered during the extraction to prevent acid vapour diffusion and replaced air bubbling with ultrasound to reduce the extraction time thus increasing sample handling capacity.

The system consists of a Zymate XP robot, three sample racks (36/pads/rack), a Mettler titrator, extraction bath, ultrasonic homogenizer, three pumps, two balances and a waste container. It provides more than 98% recovery. The extraction using ultrasound requires only 3 min while the current system 8 min. One extraction bath in this new system proved superior to or at least equal to three baths using air bubbling. In conclusion, the new system enhanced our capability of AGM assay and solved the corrosion problem.

### Automation of the Rose Gottlieb method for fat determination in dairy products using an expert system

*Alan R. Matheson and Patrick Otten, Alphatech Systems, Auckland, New Zealand*

The Rose Gottlieb test is the internationally recognized gravimetric method for the determination of fat in a wide variety of food products. It is extensively used in the New Zealand dairy industry for the calibration of infrared testing equipment and for the customer acceptance test.

The hardware and software used to automate this time-consuming and tedious assay were described. The sequence of operations is determined by an expert system rather than a scheduler. The expert system regularly queries the status of the automated equipment and then issues appropriate commands to the robot controller, after consulting a rule set describing the assay procedure. This still relatively novel approach to laboratory robotics control results in a high degree of concurrency and the ability to adapt to incoming samples with varying assay protocols.

### Automation of multiple parallel solid phase synthesis: reproducing the scope of synthetic transformations on solid phase without compromise

*James B. Campbell and Richard A. Wildonger, Zeneca Pharmaceuticals, Inc., Wilmington, DE, USA*

A collaborative effort between Zymark Corporation and Zeneca, Inc. (USA) during the past year has produced a novel, high capability automated parallel solid phase synthesis instrument as the central component of an integrated workstation-based system. A fundamental objective in the collaboration was to ensure that chemistry would guide the decision of the automated platform to enable reproduction of the manual operations employed by the practicing solid phase synthesis chemist as precisely as possible. The instrument resulting from the collaboration has incorporated several unique functional capabilities that mirror, and to a certain extent, enhance some of the process operations involved in solid phase synthesis. Notable among these capabilities are three methods of liquid reagent and solvent transfer which can function independently or in concert. These include:

- Parallel delivery and clearance (multiple channel).
- Serial delivery (single channel, closed architecture).
- Robotic delivery (single channel).

Other capabilities include access to broad temperature ranges for both heating and cooling, the non-aggressive agitation of resin, and the strict maintenance of an inert environment. Examples of representative synthetic transformations that were performed on the synthesizer were presented and compared with manual methods of synthesis. Also, advantages of the modular platform and integration with other automated workstations were presented.

### Progress toward the implementation of an integrated workstation-based multiple parallel solid phase synthesis system

*Richard A. Wildonger and James B. Campbell, Zeneca Pharmaceuticals, Inc., Wilmington, DE, USA*

In response to the burgeoning advances in the high throughput synthesis arena, and in consideration of our requirements, we sought to acquire, or develop a workstation-based synthesis system that would meet our current needs in supporting solid phase parallel synthesis and would be amenable to modification and expansion to meet future needs. Thus, we set out to automate selected sub-processes associated with multiple parallel solid phase synthesis. These sub-processes included diversity reagent preparation, synthesis (reagent addition, mixing, and washing), off-line incubation and product cleavage. As the heart of the system, the synthesis workstation required ambitious specifications in order to be able to accommodate the broad range of chemistry we wished to investigate. We fully recognized that with these high expectations, we had defined an exceedingly complex instrument.

Against these criteria, no currently available commercial product measured up to our expectations or requirements. Therefore, a collaborative effort between Zymark Corporation and Zeneca Pharmaceuticals (USA) was established to develop an instrument. In this collaboration we were guided by the belief that the chemistry should define the automated platform rather than trying to adapt the chemistry to an existing platform. During the past 18 months a high capability automated MPS synthesis instrument that incorporates features of both plumbed/valved and robotic machines has been produced. This instrument is unique in that it exemplifies three modes of reagent addition, namely plumbed-serial and plumbed-parallel, and robotic-serial. Furthermore, spent reagents, impurities and soluble byproducts are removed by filtration using positive pressure differentials rather than vacuum thus avoiding atmospheric contamination. Additional features of this instrument were discussed and details of its performance to date provided. Progress toward implementing the other workstations in this system was outlined and some directions for future system expansion were proposed.

### Evolutionary design of a combinatorial chemistry system for late stage lead optimization

*Harold N. Weller, Bristol-Myers Squibb Pharmaceutical Research Institute, Princeton, NJ, USA*

Combinatorial chemistry has proven to be a powerful tool for lead generation in drug discovery programmes. Application of automated synthesis methods to later stages of lead optimization has received less attention, largely due to the difficulties associated with characterization and purification of large numbers of samples. This presentation described the evolution of a combinatorial synthesis system designed for lead to optimization in the late stages of drug discovery programmes, including modules for synthesis, analysis and purification. Evolution of these modules in an environment where the need to produce new compounds immediately is balanced against the need to develop optimal automated procedures was described.

### Process integration of automated synthesis stations: 'gluing' the parts together

*Michael Routburg, Al Washington and Jeff Pan, Abbott Laboratories, Abbott Park, IL, USA*

In the development of automated systems for parallel synthesis, the glue that is necessary to piece together the separately automated tasks into an integrated process has both hardware and software components. The following list, while not complete, represents the bulk of the required interfacing.

*Hardware glue components*:

- Changing the product from liquid to dry state, and back again.
- Transferring the product from one vessel to another.
- Distributing accurate portions of the product from one container to another.
- Identifying the containers.

*Software glue components*

- Tracking single samples and/or purified fractions.
- Combining selected samples into groups for ease of processing.
- Identifying and accounting for exceptions (for example, insolubles, dual tube fractions, and by products of the reaction unrelated to the desired product).
- Monitoring the process.
- Archiving data to a central data repository.

These hardware and software components are solution independent, and will most likely need to be addressed in any parallel synthesis automated system integration regardless of the format of the automation being integrated. This paper discussed the various parts of the glue, and then addressed the approach Abbott used for the software and hardware integration of the overall automation.

### Robotic solid phase extraction and GC/MS analysis of THC in blood

*William Stonebraker, State of Utah, Department of Health, Salt Lake City, Utah, USA*

The ever-increasing number of DUI arrests worldwide necessitates an accurate, sensitive and easily automated analytical method for analysing THC (tetrahydrocannabinol) in the blood of marijuana smokers. The method developed by our laboratory utilized Zymark Rapid-Trace Robotics and SPE (solid phase extraction) to extract the THC and THC-COOH from blood samples submitted by law enforcement agencies in DUI arrests. After extraction, the samples are derivatized with TFAA (trifluoroacetic anhydride) and then analysed using a VG Instruments Trio-1 GC/MS equipped with NCI/SIM (negative chemical ionization with single ion monitoring). The molecular ions of the two compounds are monitored in this analysis, and a deuterated internal standard is added for quantitation.

The method is based on a manual method that has been performed for several years by the laboratory and allows a maximum of 60 samples per run. The limit of detection (LOD) is equal to or superior to the manual method. The analysis requires approximately one half the time necessary for the manual method from the beginning of sample preparation through extraction/derivatization and GC/MS data reduction to the final report.

### Application of a BenchMate™ biological monitoring method for the chlorophenoxy acid herbicides 24D, dicamba, dichlorprop, 245TP, 245T, and 24db in urine

*Ken Brown, C. A. Striley, C. D. Lorberau, and C. J. Hines, National Institute for Occupational Safety and Health, Cincinatti, OH, USA*

A poster entitled 'BenchMate™ assisted chlorophenoxy acid herbicides urianalysis method for biological monitoring of exposed field applicators' was presented at ISLAR '96. This poster described a method developed around the use of an automated workstation. Most of the analytical methods developed in our laboratory are for small batch sizes of novel analytes, which are not commercially available. Was this type of laboratory automation amenable to our laboratory? How did the method perform? What were some of the maintenance and repair issues encountered during the study? Did automation reduce the handling of urine and toxic reagents and analyst stress resulting from monotonous repetitive tasks?

Since the initial presentation, this method has been applied to two sets of human urine samples, $N \approx 50$ and $N \approx 600$. For processing, each analysis batch contained 30 samples; this included eight calibration urines, three QC urines, one interbatch split, one intrabatch split, two blank waters and one spiked water. This high percentage of quality control samples left room for only 17 field samples per batch. However, tracking the quality control samples over this study allowed us to characterize this method for accuracy, precision, carryover, and sample throughput. The performance of the BenchMate™ Workstation, HP 6890 Gas Chromatograph, and the method was monitored during the analysis of these samples.

The BenchMate™ performance was monitored by tracking the output.dat file from each batch which contained a mass audit record of each sample preparation step. For each mechanical step out of control, concurrent repair and maintenance logs were examined. The performance of the GC instrument was monitored using an internal instrument performance standard (IIPS) in each sample. The IIPS added after sample preparation to the extract was used for the determination of chromatographic efficiency, retention time, and sensitivity (mV/ng) for each sample. An internal standard (IS) added to the derivatization efficiency and precision. Method performance was also characterized for each batch by monitoring blank water, spike water, QC urine, interbatch split field, and intrabatch split field sample analyses.

In order to more fully evaluate this automated GC procedure, samples were assayed using other analytical methods for comparison. One batch of samples was simultaneously analysed by an automated immunoassay method and another batch by isotopic dilution high resolution MS using C13 24D and deuterated dicamba.

In summary, the BenchMate procedure was precise and sensitive. It also reduced sample handling by the analyst and compared favourably with results of other analytical methods. These results suggest that the automated procedure is the method of choice for unique biological monitoring methods.

### A versatile, open-access, high-throughput microplate format bioanalytical robotics system

*Glenn A. Smith, Jimmy Bruner, John R. Alianti, GlaxoWellcome Inc., Research Triangle Park, NC, USA*

Recent advances in automation, programming and microplate formatted bioanalyses have facilitated the implementation of novel bioanalytical robotics systems. These latest systems combine the main advantages of traditional robotics systems (i.e. versatility and fully automated assays) and workstations (i.e. ease of use, reliability and productivity), as well as offering a few new advantages:

- Each system can support the link multiple applications without reconfiguration or additional programming. Furthermore, the systems have been created to support not one but most types of bioanalytical extraction techniques in a 96-well format. These include ultrafiltration, protein preciptation, liquid/liquid and solid phase extractions.
- Custom Visual Basic software allows the users, even those with little or no Zymark and/or Tecan experience, to intuitively set up a new application or select and run a previously created application. The software allows the user to define, automatically link and subsequently execute their assay, even when the system is actively running another application.

The systems are each comprised of a Zymate XP robot, a Tecan RSP 8051 liquid handling system for the transfer of samples, a reagent addition station (RAS) equipped with dual 12-port solvent selection valves for all liquid additions, a microplate vortex station, a 96-well evaporation station, a Sagian plate sealer, a temperature controlled carousel for the storage of all microplates and extraction devices (i.e. SPE blocks and filtration plates), and most importantly, a custom microplate centrifuge which serves as an extraction station for all supported applications.

### Advances in membrane SPE for high throughput pharmaceutical bioanalysis

*David Wells and Li Song, 3M I&C Sector Research & Development, St Paul, MN, USA*

Applications of membrane and solid phase extraction in 96-well microtiter plate format were presented for the analysis of drugs in biological fluids. The 96-well plates contain particle loaded membranes which are composed of chromatographic particles tightly held together within an inert polytetrafluorethylene (PTFE) matrix (90% particles: 10% PTFE, by weight). The membrane SPE technology, as a result of its small bed volume, allows analyte elution in a very small solvent volume (compared when processed with small volumes of mobile phase compatible solution). The injection can be made out the very same collection plate when configured with an extraction method, together with elimination of the eluate evaporation step, to contribute to fast processing times. The processing speed complements the high throughput conventional LC/MS analysis. Automation of the extraction method is accomplished by a variety of liquid handling workstations which are programmable and offer semi-automated processing of 96-wells' plates. Each of these automation options was presented in detail. The automated membrane SPE system provides the solution to the sample preparation bottleneck now facing bioanalytical chemists.

### Development and validation of an automated method for the analysis of 3,3′,4,4′-pentachlorobiphenyl (PCB-126) and 2,3,4,7,8-pentachlorodibenzofuran (4-PeCDF) in rat tissues

*Ed Psurny, B. Harritos, K. Carrico, B. Burback, J. White and S. W. Graves, Battelle Memorial Institute, Columbus, OH and C. S. Smith, National Institute of Environmental Health Services, Research Triangle Park, NC, USA*

An automated method has been developed, validated and used for the analysis of PCB-126 and 4-PeCDF in serum, fat, lung and liver tissues from Harlan Sprague Dawley rats utilizing the Zymark BenchMate™ Workstation and gas chromatography/high resolution/mass spectrometry (GC/HR/MS). The method was developed for the National Toxicology Program's Toxicology Equivalence Factor Dioxin Initiative (TEFDI). The large number of samples expected to complete TEFDI required a method that was rugged, precise, efficient and cost effective. Most current methods in the literature are cost, labour and time intensive. Therefore, a method utilizing automated solid phase extraction was developed for these compounds. The final 4-PeCDF tissue cleanup consisted of saponification, hexane extraction, and automated solid phase extraction using a silica gel cartridge followed by a florisil cartridge. The final PCB-126 tissue cleanup consisted of saponification, hexane extraction, and automated solid phase extraction using silica gel and activated carbon cartridges. Samples are then analysed by GC/HR/MS. The range of the assay is from 5 to 1000 pg/g. Over 1500 toxicokinetic study samples have been analysed by these methods to date.

### A high throughput analysis of a novel drug candidate in human plasma using a fully automated on-line sample preparation system

*L. Lin, J. Y.-K. Hsieh, B. K. Matuszewski, and M. R. Dobrinska, Merck Research Laboratories, West Point, PA, USA*

Automation of sample preparation and analysis not only increases sample throughput but also reduces sample turnaround times. It is the most cost-effective way to meet the demanding objective of the ever-increasing workload faced by bioanalytical laboratories today. Full automation, in which the entire analytical procedure from sample preparation, extraction, data analysis to report generation is automated, has been the ultimate goal of many laboratories. Recent advances in the design of laboratory automation equipment provide powerful and flexible means for achieving full automation. This paper described a high performance liquid chromatographic (HPLC) system coupled with a fully automated solid phase extraction (PROSPEKT®) unit, a fluorescence detector, and a data processing system. The solid phase extraction tasks performed by the PROSPEKT® unit include on-line sample loading, cartridge conditioning, cartridge washing, analytes eluting, HPLC injection, data analysis, and report generation. A selected application (quantification of a novel drug candidate in human plasma) was used to illustrate the performance and benefits of this fully automated system. Validation and stability data from the analysis of spiked and subject plasma samples were also presented.

### LC-MS/MS method for the analysis of salbutamol in human plasma and urine using automated solid phase extraction (SPE) for sample preparation

*T. Sauter, J. Schmid and D. Scheutze, Boehringer Ingelheim Pharma Deutschland, Biberach an der Riss, Germany*

A sensitive method for the analysis of the $\beta_2$-agonist salbutamol in human plasma and urine was developed and validated. After addition of the internal standards [$^2H_9$] salbutamol, salbutamol was extracted from plasma or buffered urine by automated SPE procedures on a Zymark RapidTrace™ Workstation. Chromatography was achieved on an analytical C18-HPLC column with gradient elution. Salbutamol was quantified by MS/MS with electrospray ionization.

The salbutamol sulphate metabolite was separated from the salbutamol by chromatography (plasma) or the SPE procedure (urine). The calibration curves of undiluted samples were linear over the range of 15.0–4000 pg/ml for plasma and 0.250–100 ng/ml for urine. Assay validation sample measurements showed inaccuracies of the quality control samples of 2.8–0.4% for plasma and 2.4–6.4% for urine. Maximum assay imprecisions were 5.9 and 5.4% for plasma and urine samples, respectively.

Maximum carry-over effects obtained in the chromatographic system were in the range of 0.3%. Carry-over effects in the RapidTrace™ Workstation measured after preparation of samples spiked with 100 ng salbutamol/ml urine were below 0.1%. The total recovery after automated SPE was 79.1–81.6% in the concentration range of 100–1200 pg salbutamol/ml plasma. For urine, it was 70.5% and 62.1% at concentrations of 2.00 and 100 ng salbutamol/ml, respectively. The method was successfully applied for the analysis of about 3000 samples from clinical trials.

### Development and routine bioanalysis of a novel drug substance using automated 96-well solid phase extraction and LC-MS-MS

*S. R. Bessant and D. R. Lachno, Lilly Research Centre, Windlesham, Surrey, UK*

A rapid, robust and sensitive method was required to routinely determine levels of a novel drug substance in human plasma to support early phase clinical trials. A Canberra Packard Multiprobe pipetting robot with SPE functionality was the instrumentation of choice for both method development and routine analysis. Preliminary work identified 3M Empore™ discs as the most suitable extraction medium with 96-well capabilities and the 3M vacuum manifold gave greatest vacuum efficiency.

Initial development problems included carry-over between wells, inconsistent extraction across the plate and blocked wells. These issues, once identified, were relatively easy to overcome with simple adjustments to the extraction procedure and the vacuum pump used. The method was completed by development of an LC-MS-MS assay which enabled rapid analysis of extracts using a Sciex API III+ instrument in the multiple reaction monitoring mode using a deuterated analogue of the drug substance as the internal standard.

The most obvious advantage of this method is speed where a number of factors are contributory. SPE using the 96-well format on the Multiprobe and the speed of the LC-MS-MS analysis are obvious time-savings but additional time is saved by the reduction in sample handling and pipetting. Data quality is also improved as the chances of human error and inconsistencies are reduced, making this method not only more efficient in the originating laboratory but more compatible with method transfers.

### An efficient solid phase extraction of a novel drug candidate from plasma using Empore™ disks in a 96-well format and application of the method for assaying samples from human pharmacokinetic studies

*R. S. Mazenko, E. J. Wolf, J. Hsieh, B. Matuszewski, Merck Research Laboratories, West Point, PA, USA*

Our laboratory is investigating a variety of methods to increase the throughput of clinical sample analyses. As part of this effort, the applicability of using Empore™ disks configured in a 96-well format for the extraction of a novel drug candidate from human plasma samples was evaluated. The sample preparation portion of a previous developed method for the determination of a proprietary non-polar drug candidate in human plasma using HPLC and fluorescence detection was successfully modified to use the extraction plates rather than conventional individual solid phase extraction (SPE) columns. The ability to elute the analytes of interest from the extraction plate with a small volume of solvent was found to eliminate the need to evaporate extracts and reconstitute the samples prior to analysis. Consequently, sample throughput could be increased by 50% as compared to the conventional solid phase extraction procedure. The modified assay was validated over the concentration range of 5 to 400 ng/ml. Replicate analyses ($n = 5$) of spiked plasma standards from 5 to 400 ng/ml yielded coefficients of variation less than 2.3%, and accuracies within 3% of the nominal concentrations. Details regarding the conversion of the assay to the 96-well format were presented. The method was successfully applied to the analysis of plasma samples from several pharmacokinetic studies. Additionally, the applicability of using an automated liquid handling station to perform the extraction of clinical samples with the Empore™ plate was described.

### An integrated information system for drug disposition data

*Wade J. Adams, Paul A. Bombardt, John D. Hosley, Kathleen S. Chapman, Donald P. Dame, Chau H. Mai, Nancy J. DeBliek, Chris J. Glass, Dianne C. Hollenbeck, Phillip M. Sundman and Dean W. Knuth, Pharmacia & Upjohn, Inc., Kalamazoo, MI, USA*

An integrated information system has been developed by Pharmacia & Upjohn, Inc., for the interactive analysis of the large amounts of data typically generated in single- and multiple-dose drug disposition studies. The system utilizes IBM PC compatible workstations in conjunction with a Harris NH4402 super minicomputer and PENelson 900 series chromatographic interfaces for real-time acquisition and processing of chromatographic data, inhouse developed software for importing chromatographic data from remote research facilities electronically using a standardized data format, an IBM 310 P133 server for acquisition, processing and storage and analytical balance and scintillation counter data using the commercially available DEBRA System (Version 4.2, LabLogic System, Sheffield, UK), and an IBM 3090 mainframe computer for protocol specific integration, storage, pharmacokinetic and statistical analyses, and archival of data.

Chromatographic data from automated gas and liquid chromatographs equipped with a variety of detectors, including mass spectrometers, are acquired on the Harris NH4402 and processed to calculate drug/metabolite concentrations for quality controls and unknowns from standards data using the in-house developed UPACS Chromatography System. Following evaluation of data for chromatographic run, standards, controls and unknowns data are transferred electronically from the UPACS Chromatography System or from remote research facilities to the in-house developed ADME System resident on the IBM 3090 using standardized formats. The data are stored by study protocol in an SQL/DS data base. Raw data for each UPACS Chromatography System analytical run can then be achieved on the Harris NH4402, and the data from remote research facilities can be retained on the IBM 3090.

In a similar manner, drug dose, body weight and drug equivalents data are transferred electronically from the DEBRA System to the ADME System following evaluation of the data for each run. The raw data can then be archived on the IBM 310 system. The ADME System can be used to edit the integrated data for a protocol, calculate preliminary pharmacokinetic parameters, generate tabular reports and plots, and allows the user to selectively extract and transfer data using standardized formats for more extensive pharmacokinetic and statistical analyses of the data or the preparation of user-customized reports. This integrated information system increases the reliability of data by eliminating data transcription, provides audit trails of edited data, and greatly reduces the time and effort necessary to analyse and report data from single- and multiple-dose drug disposition studies. In addition, the system is GLP compliant.

### PhASSETS®, an automated extraction system for fast method development and production in analysis of trace levels of drugs in biological fluids

*Carl Dourambeis, Rene Michaud, Daniel Dorval and John Burrows, Phoenix International Life Sciences, Inc., Montreal, Canada*

PhASSETS® (*Ph*onenix *A*utomated *S*imple *S*ample *E*xtraction *T*reatment *S*ystem) is a novel technology, developed at Phoenix International used to extract trace levels of drugs from biological fluids. The PhASSETS® system includes a proprietary, pretreatment, multilayer, extraction cartridge accompanied by a robotic processor. This system allows the development chemist the ability to perform multiple extractions in a single pilot run. PhASSETS® uses thin-film liquid/liquid theory. Up to four extraction solvents, four internal standard analogues, and a variety of specially prepared cartridges may be used together to quickly develop methods. Manual methods in comparison, may take up to two weeks to obtain the same results obtained by one PhASSETS® pilot run. Key features of this bioanalytical extraction system include the ability to extract large volumes of samples to be used in various detection laboratories (HPLC, GC, GC/MS, LC/MS, etc.). Through key examples, the method development technique used with PhASSETS® as well as the scope of the applicability of this novel system was presented.

### High throughput extraction of basic and polar drug compounds from biological matrices with a novel polymeric solid-phase sorbent in a 96-well format

*Judy L. Carmody, Yung-Fong Cheng, Dorothy Phillips, Pamela Iraneta and Uwe Neue, Waters Corporation, Milford, MA, USA*

Historically, sample preparation has been the bottleneck in laboratory productivity, especially with the onslaught of LC/MS/MS. Rapid and reproducible quantitation of classically difficult, basic and polar drug compounds from biological matrices has, in the past, been a challenging, time-consuming endeavour. Necessary sample preparation techniques used to concentrate or clean-up matrices before HPLC, GC or LC/MS analysis of basic compounds have proven to be either tedious and labour intensive or irreproducible. Presently, the most commonly used technique for high throughput sample preparation is reversed-phase solid phase extraction in a 96-well format. The sorbent generally utilized is porous silica surface-bonded with $C_{18}$. The major disadvantage to employing this type of system with basic compounds is that the silanols on the silica surface can deleteriously affect the recovery of the basic compounds. The surface silanols interact through ion exchange with basic compounds, such as Doxepin. This interaction prevents complete elution of basic compounds and results in low, variable results. With polar compounds, such as Procainamide and Ranitidine, the analyte/sorbent interaction is low resulting in breakthrough of the polar analyte. This subsequently, yields poor, irreproducible recoveries. Another notable disadvantage to using traditional silica-based reversed-phase sorbents is that the wettability must be maintained through the tedious manipulation of stopcocks. The capacity of $C_{18}$ is severely compromised if the sorbent runs dry.

This paper showed how the sample preparation bottleneck has been eliminated. Highly reproducible and uncomplicated SPE methods were developed for these types of basic and polar drug compounds using a novel polymeric solid-phase sorbent, Oasis™ HLB, in a 96-well format. Recoveries greater than 90% and reproducibility less than 5% RSD ($n = 96$) for basic antidepressant and polar drugs were realized. Most importantly, because Oasis™ HLB is a water wettable polymer, these results were achieved without concern as to whether or not the sorbent ran dry.

### The robotic manufacture of a non-disintegrating polymeric capsule for controlled release applications

*A. R. DeNoto and A. G. Thombre, Pfizer, Inc., Groton, CT, USA*

A robotic process was developed to manufacture non-disintegrating polymer capsules for controlled release applications. The capsules were made by a phase inversion process—the membrane structure was precipitated on a mold pin by dipping it in a coating solution followed by quenching. The coating solution was a polymer–solvent–nonsolvent system that yielded an asymmetric membrane wall, i.e. a wall composed of a thin dense region supported by a thicker porous region. The final dosage form was assembled by filling the capsule with a drug–excipient mixture and then sealing the seam of the capsule body and cap.

A Zymate II robotic system (Zymark Corporation, Hopkinton, MA) was programmed to perform the unit operations of dipping, withdrawing, spinning, quenching and drying the capsules. The manufacturing set-up consisted of the robot at the centre and the following elements arranged in a circular fashion within the reach of the customized robot-arm:

- A line-feed for the fixtures with mold pins.
- The dip-bath for the coating solution with a pneumatically operated cover.
- The quench bath capable of holding a maximum of 30 fixtures.
- A blower to get rid of any excess liquid remaining on the capsules.
- Drying rails.

The robot was programmed to sequentially process six dip fixtures. The robot arm picked up a dip fixture from the line feed and positioned it above the dip solution tank. The pneumatically operated cover was automatically opened and the dip fixture was plunged slowly into the coating solution and withdrawn over 12 s. After withdrawal, the fixture was rotated twice in opposing 360° turns. It was then moved to the quench solution bath. This cycle was repeated with the remaining dip fixtures. After 15 min in the quench bath, the robot was programmed to transport each of the fixtures over a 10 s air blast and park them on rails for a 30 min drying step. The fixtures were then removed from the rails from the manual stripping, trimming, and joining operations.

The robotic process was versatile which proved extremely useful during the process development and optimization stage. Capsules of consistent quality were manufactured in quantities typically required for formulation development and for providing supplies for clinical, toxicity, and stability testing.

*Acknowledgements*

The authors acknowledge the engineering support given by Alam Curtiss and George Butler.

### A Bohdan automated workstation for microbial identification sample preparation

*Martha H. McDaniel, Nancy J. Hodges, Robert L. Dinkel, Robert L. Ellington, and Michael L. Rutherford, Eli Lilly and Company, Indianapolis, IN, USA*

In the last several years, regulatory agencies' expectations for environmental monitoring have increased dramatically, not just for parenteral operations, but for all pharmaceutical manufacturing operations. As a result, there is an increased need for microbial identification. Classical microbiological techniques, such as the API System and bioMerieux Vitek System, have been used for years to identify various microorganisms. However, these techniques can be very time-consuming and/or expensive, and do not provide the high throughput capabilities necessary to meet the growing demands for microbial identification. Several newer alternatives have been developed which can address the lower cost and potential higher throughput needs, such as the Microbial Identification System (MIS) Whole Cell Fatty Acid Analysis by Gas Chromatography by MIDI. This technique uses peak naming and pattern recognition algorithms to identify fatty acid extracts of microorganisms processed on a Hewlett-Packard gas chromatograph. The preparation of the extracts is divided into five basic steps: harvesting, saponification, methylation, extraction, and washing. The latter four steps require various sample processes including the addition of reagents, vortexing and mixing, heating and cooling, and liquid phase extraction. Consequently, this extraction process can be rather tedious and labour intensive, especially in a high volume situation.

In order to meet an increased need for sample capacity, the authors worked with Bohdan Automation, Inc., to develop a compact benchtop automated workstation to perform the saponification, methylation, extraction, and washing steps of the microbial sample extraction. Through the adaptation of novel combinatorial chemistry/reaction block approaches to switch from slower serial to more efficient batch modes of operations, it was possible to automate this sample preparation. A description of the assay methodology along with the workstation components, features, and capabilities was presented. Comparison data for the automated versus manual methods was also presented.

### A Gilson 215 multi-probe liquid handler: a versatile system for under $26 000

*Terry L. Stinger and Michael L. Rutherford, Eli Lilly and Company, Indianapolis, IN, USA*

Since its introduction several years ago, the Gilson 215 Liquid Handler has proven itself to be a versatile, large capacity, septum-piercing liquid handler for fast, safe, and efficient sample preparation and transfer. This XYZ robotic system can be easily programmed to automate any number of liquid handling procedures, including combinatorial chemistry, RIA or ELISA bioassays, colorimetric assays, quality control testing, autoloading of NMR tubes, automated compound distribution studies, serum transfer and dilution, and sample preparation and injection to HPLC. Another added benefit is the 215 Liquid Handler's ability to pierce VACUTAINER™ tubes and other equivalent thick septas, an option not available on other economically priced liquid handlers. The 215 Liquid Handler's open architecture and graphical tray editor allow for a wide variety of racks and vessels ranging from microplates to test tubes to 125 ml bottles. With the emergence of high throughput screening methodology and the increased use of microplates for analyses, the speed and throughput of a single-probe liquid handler can quickly become a limitation, despite a low cost. Several multi-probe liquid handlers are available today, but the higher cost for these systems are more difficult to justify, especially for laboratories with low sample volumes and limited resources. For this reason, the authors worked with Gilson to redesign their single-probe 215 Liquid Handler to provide multi-probe capability, while maintaining the economical price. As a result of this collaborative effort, this Gilson 215 Multi-Probe Liquid Handler with up to eight probes is now commercially available for under $26 000.

A description of the components, features, capabilities and hardware limitations of the Gilson 215 Multi-Probe Liquid Handler was presented. Several applications were described to demonstrate the versatility, programmability, and benefits of this low cost multi-probe liquid handler.

### Automation of the sterility test

*Vanni Visinoni, FKV, Sorisole (BG), Italy*

The norms regulating the execution of the Sterility Test are under revision, but it is expected that the incubation time will increase to 14 days and the retest opportunity will be reduced. The cost of secondary contaminations (false positive) is going to impact drastically on the balance of a pharmaceutical company, not only because of the value of the rejected product, but also as loss of image, the need to modify the production schedule, etc. Since 90% of the false positives are generated by the operator, a reduction of these cases could be achieved by the use of a sterile robot performing the test inside a clean room. This approach will also allow a reduction of the time spent by the operator inside a difficult environment like the clean room.

An automated system is now available, based on the use of a modified Steritest Kit, engineered by Millipore and on a robotic arm provided by Zymark Corporation. The automated system allows the execution of up to 40 batches in a single run for a duration of around 20 h. Only 30 min of the operator's time is required at the beginning and at the end of the run to load and unload the samples and the kits. The automated system is capable of handling different types of containers like vials (from 5 ml to 100 ml), ampoules (from 2 ml to 100 ml) or prefilled syringes. Samples may be of different types, like powders, liquids, bulk materials or lyophilized products. The system has been designed to allow easy decontamination as well as easy removal of all the components requiring sterilization.

### Automated methods development and optimization of a compound in medicated feed blends using a Zymark Pytechnology system

*L. C. J. Erhart, Pfizer, Inc., Groton, CT, USA*

An overview of the automated process was highlighted from a liquid chromatography (LC) perspective. The process was sequentially outlined showing the development of an analytical extraction method of a drug analyte from feed matrix. Solubility of the drug substance and polarity index of the extraction solvent is utilized to optimize resolution. The extraction efficiency is maximized to obtain 100% recovery with reduction of interferences to little or none. Assay development time is reduced by 50%. Utilizing a 486 PC, Windows, VBX Visual Basic, Easylink, DDE and Excel, a Zymark XP robot adds extraction solvent via a nine port valve and extracts the drug component. The sample is transferred to a BenchMate II Workstation for dilution, mixing and filtration. The final extract is transferred to an EasyFill via filling station prior to LC injection.

### Application of robotic dissolution of a tablet formulation to meet aggressive timelines for laboratory certification in support of key investigational batches

*Chrone Chen and Nita Patel, Pfizer Inc., Brooklyn, NY, USA*

Dissolution has become an essential test in the control laboratory certification programme for getting ready to support dosage form manufacturing in an international pharmaceutical environment. The use of a Zymate XP robot in the dissolution testing of a tablet formulation has addressed the laboratory certification schedule, labour demand, and occupational exposure posed by this potentially hazardous new drug product candidate. This robotic application demonstrates how automation can help meet business needs, achieving fast results and ensuring personnel safety.

### A robotic method for dose delivery assay for a beclomethasone dipropionate MDI

*M. O'Kane and S. Li, Schering-Plough Pharmaceuticals, Kenilworth, NJ, USA*

A robotic dose delivery assay method has been developed for a beclomethasone dipropionate MDI. The procedure uses a Zymark® robot designed to test metered dose inhalation products. The robot system was first validated to ensure that the robot would execute the program as expected. Then, the validation of the dose delivery assay procedure was performed. There were two primary phases of procedure validation. In the first phase, the robot was optimized to perform the dose delivery assay. This included the efficient rinsing of the collection vessel used to collect the samples, finding a suitable pressure to be applied to the plungers used to actuate the sample cans, and performing a primary study to determine the number of times a sample can needed to be actuated by the robot prior to sample collection. In the second phase, it was demonstrated that the robot was able to generate sample data comparable to sample data obtained using the manual procedure. The robot has the capability of assaying 45 aerosol cans. Up to a maximum of 150 vials of sample solution can be prepared for HPLC analysis.

### DMP 266 capsules: validation of automated methods using Zymark WorkStations

*J. A. Short, The DuPont Merck Pharmaceutical Company, Wilmington, DE, USA*

DMP 266 is a non-nucleoside reverse transcriptase inhibitor under development at DuPont Merck Pharmaceutical Company and is currently in clinical trials. Due to the number of strengths being developed and the large volume of release and stability testing required to support activities to NDA, automated sample preparation methods for assay, degradation products and content uniformity were developed.

The drug is formulated in capsules which presented unique challenges to our automation procedure using the WorkStation. The method and equipment problems were resolved and validation of the automated methods is now complete. The technical challenges which were overcome were discussed, and the validation results were presented.

### Modular approach to automation in a laboratory supporting dentifrice product development

*Robert E. Barron, Procter and Gamble Health Care Research Center, Mason, OH, USA*

Product development requires a different type of analytical support than does manufacturing. In a manufacturing setting, there is typically a limited number of methods run on a large volume of samples. In a product development setting, there is usually a much larger number of methods and mostly small to medium numbers of samples to run at any one time. The approach to automation in these two situations is also different. Large, high-throughput, do-it-all systems usually make sense in a manufacturing setting, whereas in a product development environment, they often don't fit. Therefore, we have taken the approach of using modular automation to automate the work processes which make sense rather than automating everything for automation's sake. We have also attempted to maintain the appropriate scale of automation for different analyses based on sample volume. An additional complicating factor when working with dentifrice is the fact that it is a thick, adhesive product with roughly 20% suspended solids. This makes sample preparation more difficult and automation hardware more complex than for many other sample types. Descriptions of the workstations employed in our work as well as an outline of our strategy were provided.

### Validation of a newly designed automated dissolution system

*Ralph Scimeca, Patrick Drumm and Douglas Judge, Novartis Pharmaceuticals, East Hanover, NJ, USA*

The validation of any computerized system is crucial for any application in a good manufacturing practice (GMP) environment. Unfortunately, this validation effort has become much more burdensome with today's complex automated systems. However, with the application of targeted challenges and quality vendor IQ/OQ/PQ documents, a high level of analytical confidence with a minimum of effort can be achieved. Using this approach, we have validated the newly designed automated dissolution module (ADM). This automated system is a bathless, single vessel, dissolution unit capable of sequentially testing tablets or capsules using USP apparatus 1.

The presentation explained the ADM processes and the authors' approach to system validation.

### Demonstrating equivalency: three essential principles in automated method validation

*Douglas Judge and Patrick Drumm, Novartis, East Hanover, NJ, USA*

Automation in analytical laboratories is capable of improving productivity levels, increasing the precision and accuracy of results, and providing greater GMP/GLP compliance. However, to obtain these benefits, the equivalency between the automated and manual methods must be demonstrated. Three important considerations in assuring equivalence are:

- *Effective experimental design*: Many subtle differences may exist between automated and manual methods; however, a well designed experimental plan will identify and account for these effects.
- *Rigorous statistical treatment*: Proper mathematical analysis is essential to interpret analytical data and reach defensible conclusions
- *Rational acceptance criteria*: The evaluation criteria for the automated processes must be justifiable when compared to the validated manual procedures and the testing requirements.

This presentation explained how these principles should be applied as an essential part of the validation of automated methods.

### Automation of sample processing for NEMACUR® residues in groundwater and soil

*Shelley L. Widmer and Gregory C. Mattern, Bayer Corporation, Stilwell, Kansas, USA*

The Environmental Fate Team at Bayer Corporation, Agriculture Division is responsible for the development of pesticide residue methods and the analysis of thousands of soil and water samples annually. In order to meet the demands of shortened development time and the large number of samples, several Zymark Robotics systems were designed to process both soil and water samples from field studies required by regulatory agencies for registration of our products.

NEMACUR® is a non-fumigant chemical with systemic and contact nematicidal properties. This product is currently registered for use in the United States for the effective control of nemetaodes in certain field, fruit and vegetable crops, ornamentals, nursery stock, and turfgrasses.

Two automated analytical methods have been developed to process soil and groundwater samples containing NEMACUR® and its degradates using Zymark robotics systems. Custom RapidTrace workstations, designed to handle large volume samples, use solid-phase extraction to extract the active ingredient and degradates from sample matrixes. A Zymate XP robot is used in conjunction with the RapidTrace workstations to complete the processing. The final products are 1 ml sample extracts in autosampler vials, which are available for HPLC/MS/MS or HPLC analysis.

### Development of an automated solubilization method for quantitation of radioactivity in tissues

*K. Gibson, M. Dryzga, J. Ormand and C. Thornton, The Dow Chemical Company, Midland, MI, USA*

The Health and Environmental Research Laboratory generates kinetic, metabolism and mechanistic toxicity data that are required for product registration, and provides important insight on chemical toxicity. Qualitative and quantitative assessment of metabolism is determined by processing specimens to release incorporated radioactivity by solubilization with a strong base. The purpose of this project was to streamline the solubilization process.

A solubilization method for quantitation of radioactivity in whole tissues with tetraethylammonium hydroxide (TEAH) was developed, and the process was automated using a Zymark Zymate XP robotics system. Since implementation in March 1996, a greater than 50% reduction in tissue processing time compared to the manual method has been achieved. Data quality has also improved through uniform sample history, gravimetric tracking and interfacing of the system output into our laboratory data management processes. In addition, safety was increased through lower chemical and radiochemical exposure and improved ergonomics when compared to a manual method.

### Automated assay and analysis of gels or liquid detergent

*Nick Barrett, InnovaSystems Inc. Pennsauken, NJ, USA*

The AAAGOLD system is required to aspirate a sample of liquid detergent and load the sample into cells for the three measurements to be made: colour, viscosity and pH. The measurement instruments are commanded to initiate the measurements asynchronously, as each cell is loaded. Data are then gathered by the system until a stable reading is obtained. The fluid distribution components must be washed, dried, and made ready for the next sample. Concurrently, the robot and capper station are replacing the current sample bottle and fetching the next bottle from the sample racks. Each bottle is identified by a bar code.

The software implementation takes advantage of the multi-threaded feature of the OS2 operation system. High level functions such as control of each instrument, or the distribution system, are executived on separate threads to allow simultaneous processing of data collection, sample transport, and the GUI human interface.

An important feature of the system is the use of script files to control the fluid handling part of the system. In this way, the sample handling method may be modified by the user using a simple, human readable, command set. Script based fluid handling also allowed non-programmers to experiment with the sequence and other details of the method during development and initial test. As part of the diagnostics functions, test scripts exercise industrial switches, valves and pumps of the liquid distribution system.

A separate software program was developed to collect the data describing the sample and study, and handle the post processing of the data. The program used Java to implement a GUI for the input of formatted study information, managing study files, and printing the bar codes and generating sorted results files.

### Zymark BenchMate Workstation automates DNPH preparation of PPM aldehydes in industrial samples—II

*Stephen L. Wellons, Cheryll R. Roberts, Robert J. Gentry and Jack D. Cobb, Union Carbide Corporation, South Charleston, WV, USA*

The laboratory procedure for diluting, derivatizing and extracting the 2,4-dinitrophenylhydrazine (DNPH) derivatives of aldehydes in industrial samples takes about 15 min of labour per sample. A sample preparation Zymark BenchMate Workstation can do these steps in an equivalent manner for 1–3 min of labour per sample. In the poster presented last year, the DNPH derivatives were extracted away from the acidic preparation matrix with Solid Phase Extraction (SPE) cartridges before liquid chromatographic (LC) injection. In this poster the matrix is first neutralized and then injected directly, saving time and cost. Using the BenchMate the five main variables (pH, concentration of acid, organic solvent, mixing time and mixing speed) were optimized fairly quickly. The advantages and disadvantages of the two approaches were compared.

### Adapting a Zymark robot for extraction of metolachlor from soil

*Theresa H. Lemme, Alan Olness, and Ward B. Voorhees, USDA-Agricultural Research Service, North Central Soil Conservation Research Lab, Morris, MN, USA*

Current methods for extraction of metolachlor [2-chloro-N-(2-ethyl-6-methylphenyl)-N-(2-methoxy-1-methylethyl)-acetamide] from soil are tedious and time consuming. A procedure was developed to provide a quick and reliable automated multi-step method of metolachlor extraction from soil. A robotic solvent drying station containing anhydrous sodium sulphate was configured with a glass filter device. A custom made 27-mm OD by 20-mm ID 1-mm-thick Teflon gasket was fitted between the top and bottom portion of the filter to prevent solvent leakage. A filter support was machined from a 100 × 50 × 18-mm aluminium bar and attached to the liquid–solid station tower. A hole cut in the liquid–solid station tower allowed repositioning of the pneumatic toggle clamp. A cap closure assembly was prepared from a 38 × 13-mm Teflon cylinder and attached to the pneumatic toggle clamp. A 30-mm ID centrifuge tube holder was fashioned from Teflon and positioned on the liquid–solid station shuttle in the output position. The robot was programmed to access the new station for drying the hexane–metolachlor solution, use the pneumatic toggle clamp with the Teflon cap assembly to move the sample into a large collection tube, and rinse the sodium sulphate with the appropriate solvent to ensure removal of all the solvent–metolachlor solution. The computer was programmed to divide each large centrifuge rack, use each of the four positions in the centrifuge for four separate extractions, move the liquid–solid station shuttle in to collect the dried hexane–metolachlor solvent and use one position on a second master lab station as a solvent dilution-delivery module.

### Custom automation and the validation dilemma

*Keith A. Gang, InnovaSystems, Inc., Pennsauken, NJ, USA*

Various degrees of difficulty exist when validating custom automated systems and workstations. This poster compared the validation efforts of two equally complex automated systems, both of which perform QC testing in the pharmaceutical industry.

In the first case study, InnovaSystems was contracted to validate a previously designed custom vision automation system used for the real-time quality control inspection of MDI patient actuators. The second case study involved the validation of a bench-top workstation that automatically performs a user defined sequence of spraying and weighing of nasal spray pumps. This spray and weigh workstation, InnovaSystems' Automated Nasal Spray Pump Delivered Volume Test Station, is delivered with validation documentation.

InnovaSystems has been involved with the validation of custom and 'off-the-shelf' automated systems for the pharmaceutical industry. InnovaSystems has validated various automated systems and automated laboratory equipment provided to its clients by third parties.

A comparison of the following issues with respect to the influence that a validation provider has on them (i.e. validation provided by the manufacturer as part of the life cycle of the system versus validation provided by a third party on an already designed system) was made for the two case studies. The following issues were described as they are perceived by the end user of the systems:

- Validation cost.
- System cost.
- Quality.
- Schedule.
- Maintainability.
- Usability.

### Customizing your workstation's controller

*Simon Smith, SmithKline Beecham Pharmaceuticals, West Sussex, UK*

With the acceptance of laboratory automation within the industry, the move to improve its user interface has emerged. Four years ago, we were all delighted when a new workstation actually processed a single sample into a final solution for analysis. Now, that automated process is taken for granted. As a result, other ways to improve the current laboratory automation are being sought. The ultimate vision and inevitable goal of this new wave of development is to produce a system where a tablet can be put into the front end of an instrument and the final result posted to a LIMS system for verification or printed out for the operator to take away.

As a first step towards the ultimate analysis instrument, the on-line automated checking of current equipment performance has been developed within our laboratory. Initially concentrating on system calibration and the formatting of the gravimetric data, the progression towards final results calculation can be seen. Remote operation of the automation systems has also been successfully achieved over a PC/PC link via modem. The major hurdle being an interface with the chromatography data handling system.

There are two options for the ultimate analysis system, the first being to use the system's controller to perform the calculations after transferring any necessary data from the chromatography system. The second option is to pass any gravimetric data to the chromatography data handling system and have it perform the calculations. The variations in data handling software on the market today do not make this an easy task for an automation vendor. For this reason, the first wave of the ultimate analysis systems will be developed in house.

With the evolving use of the Internet, all robotic systems could be programmed to add their errors into a globally accessible database. This pool of information would naturally be covered by appropriate security and would allow the manufacturer to monitor each geographical area enabling provision of the required service and support infrastructure. Users could also, where permitted, see which other laboratories were experiencing similar problems.

After achieving these goals, what next? Totally remote operation? Will operators on Mars be running the laboratory robot systems down on Earth?

### SEPBOX—automated isolation of natural products

*Kai U. Bindseil and Ralf God, AnalytiCon AG, Berlin, Germany*

Natural products of plant, microbial and animal origin are still high in demand as starting materials in screening for new biologically active compounds. However, isolation of pure substances from complex extracts is often tedious, time-consuming and hence an expensive process. To perform drug discovery in a more efficient way a fully automated chromatographic device was developed. This SEPBOX system is based on a skilful combination of HPLC and solid phase extraction (SPE) technologically realized as an HPLC/SPE/HPLC/SPE coupling. By this technique, fractionation of an extract into 100–300 fairly pure compounds is possible within less than 24 h. In this way, SEPBOX guarantees an efficient provision of natural compounds for high-throughput screening. Examples for the automated fractionation of plant and microbial extracts were discussed in detail.

### Development of a method for accurately and rapidly screening tight-binding enzyme inhibitors

*Martin Wong, Duan Putnam and Grant Carr, Biochemistry, Arris Pharmaceutical Corporation, S. San Francisco, CA, USA*

The accurate determination of the affinity of tight-binding enzyme inhibitors, with Ki values comparable to the enzyme concentration used in the assay, has not been routinely needed in the pharmaceutical industry until recently. As our enzyme inhibition programmes progressed, the assay lab was faced with the problem of measuring tight-binding inhibitors synthesized by our chemistry divisions. Concurrent problems were the need to minimize the turn-around time for results and to have a method in place that anyone in the lab could use. We have successfully developed and implemented a multistep protocol for determining the apparent Ki of tight binding inhibitors for several of the enzymes we routinely assay. In this poster the authors presented the primary testing method, where the inhibitor is quantitatively identified, and the steps of the secondary assay, wherein Ki app. is accurately determined.

### Building a new integrated corporate HTS environment

*Plamen Petrov, Ian Yates and Mark Divers, Astra HTS Centre, Lund, Sweden*

During the last year Astra has established and successfully turned into production a new corporate HTS Centre. The unit is designed to serve projects from the whole international Astra research organization, and also to house the HTS compound library. Keys to the rapid success of the facility are the tight integration between biological screening and compound handling, the high degree of automation and automated data management and the freedom to build a system from the best or most pragmatic options available on the market.

Our automation equipment currently includes two Zymark robotic systems and a Tecan Genesis RSP. One of the Zymark robots works as a dedicated liquid handling system, while the other is a screening system capable of running isotopic, fluorimetric and colorimetric assays. All microtitre plates are barcoded and tracked in a database (ActivityBase from IDBS, UK) during their whole lifecycle along with the experimental data. Hits are extracted from the source plates using the Tecan processor for further testing.

A further robotic system (from Thurnall, UK) for automated screening is currently being installed. It will be closely followed by an automated liquid store system (Thurnall) which will house the compound source and replicates and will eliminate all manual work in plate preparation and sampling handling.

The system is currently operational at genuine high throughputs with 96-well technology, but is well-placed to exploit other formats, new technologies and higher capacities. This is partly due to the choice of instrumentation and design, but mostly because of the well-integrated nature of the Centre, combining both the compound library and screening activities, linked by common data and automation strategies.

### The Microbial product library for high throughput screening at Biosearch Italia

*Daniel Losi, Angelo Borghi, Linda Cavaletti, Frederica Sponga, Ameriga Lazzarini, Marco Monti and Flavia Marinelli, Biosearch Italia SpA, 34, Gerenzano, Italy*

Microbial diversity is an important source of new pharmacologically active molecules, novel molecular templates for target-oriented combinatorial chemistry, as well as new biocatalysts. The exploitation of microbial diversity requires the access to geographically diverse sample sources and to different ecological niches, the employment of enrichment and/or selection methods for the isolation, and the development of a high-quality process for the preparation/use of samples in high-throughput screening (HTS).

The Biosearch Italia microbial collection contains approximately 22 000 microorganisms represented by 27% streptomycetes, 24.5% fungi and 48.5% actinomycetraspora, Actinoplanes. From 1960 to the present, thousands of strains had been isolated per year, but only those showing peculiar taxonomical or metabolic traits have been maintained in the collection. Today, the microbial diversity is continuously enriched by combining propriety isolation methods and selective media and by isolating from different sources such as soils, leaf litter, marine and lake sediments and plants.

A microbial product library is currently being generated by fermenting strains in multiple media to enhance the expression of microbial diversity. Samples are prepared by resin absorption/elution of the fermented broths, dispensed in 96-wells microplate format and ready to be used in HTS. The process of sample preparation is completely automatized by using a robotic station based on Zymark technologies and customized by FKW (the Zymark Italian subsidiary). This robotic station is able to work up to 320 fermentation broth per day employing two different sample treatments (one in development phase), and thus to generate 640 samples per day.

The data management of the microbial collection, the microbial product library, the process for the sample generation, and the results of the tests/projects of their screening requires the development of a complex information system. A customized version of Perkin Elmer SQL*LIMS ORACLE-based data management has been developed for the integration of the standard package with new forms. All the information related to each strain in the collection has been linked with the data related to each sample (for example the producing microorganism, the fermentation medium and the sample preparation method). The results generated on the microbial sample in the HTS assays are also maintained and handled by the same data management system which guarantees the analysis and the retrieval of the data.

### High throughput liquid handling at Glaxo Wellcome

*James Ballinger and Makda Mebrahtu, Glaxo Wellcome Inc., Research Triangle Park, NC, USA*

Liquid handling is a recently added function of the Compound Registration department. Until last year, the only method of filling compound requests was weighing out solid samples manually, which proved to be time-consuming and wasteful of limited supplies of research compounds. Once a small quantity of solid sample is dissolved in a diluent, then this liquid supply is sufficient for numerous requests. Handling compounds in solution is essential for the high throughput screening programme introduced at Glaxo Wellcome's US site in mid-1996.

Samples dissolved in DMSO are packed in 96-well plates and the data associated with these plates, such as volume, concentration, and plate maps are transferred into a liquid inventory system. This inventory system generates barcodes containing updated information pertaining to the aliquoting, diluting, or pooling method applied to the original plates. The tasks created in the database are then carried out physically in barcoded plates by means of a Zymark RapidPlate and Tecan Genesis. Aliquots are then distributed to designated screen sites. After testing, the compounds considered to be of interest are selected and resubmitted for further testing.

Over the last year we have provided over 365 000 liquid samples to the screening sites of R&D. In the future, we plan to address and improve aspects of liquid handling such as cold storage, stability, plate labelling and solubility, as well as to explore new automation techniques.

### Fully automated compound distribution to multiple biochemical targets

*Joan Frezza, Julie Tomlinson, and Brent Butler, Glaxo Wellcome Inc., Research Triangle Park, NC, USA and Matthew Klemp, Zymark Corporation, Hopkinton, MA, USA*

Drug discovery in the 1990s has adopted high throughput screening to increase productivity by quickly evaluating the vast numbers of compounds generated through compound libraries and other synthesis technologies. High throughput screening necessitates the replication and dilution of hundreds of thousands of compounds, which are today formatted and stored in 96-well plates. These compound plates are subsequently tested for biochemical activity against multiple targets.

In the early stages of screening, compounds were diluted and dispensed using RapidPlate and Tecan workstations operated by people; plates were labelled and sealed manually. Just dispensing compound required the work of five full-time people 10 days/month. All of this work is now fully automated.

To facilitate this process, GlaxoWellcome's Department of Molecular Biochemistry worked with Zymark Corporation to fully automate its compound-handling process. Together they engineered a system which integrates a Tecan Genesis 150, two Zymark Rapid Plates, three storage carousels and six random racks with a 3-m undertable track and a Zymate XP robot. The system's liquid handling performance is routinely verified and its compound handling processes are frequently changed to meet the changing needs of GW's biochemistry processes.

At the time of this presentation, this compound handling robot had been operational for six months and had prepared tens of thousands of compound plates for GW biochemical targets.

### Automated sample preparation for drug discovery with modified Zymark RapidTrace modules

*I. Schmid, I. Sattler, S. Grabley and R. Thiereck, Hans-Knoell-Institute, Jena, Germany*

Secondary metabolites from plants, animals and microorganisms show a structural diversity that chemically synthesized compounds can hardly reach. For this reason natural products are interesting sources for automated screening programs in drug discovery. Unfortunately, extracts from natural sources are usually complex mixtures of compounds. As the quality of the provided samples play a pivotal role in the success of high throughput screening programmes this poses serious problems. A method of sample preparation which reduces complexity and simplifies the identification of an active compound from a mixture would avoid tedious and time consuming steps in the drug discovery process.

This presentation described a new automation approach of sample preparation with eight modified Zymark RapidTrace modules. The workstation was designed and set up in co-operation with Zymark GmbH, Germany. A fast and easy-to-handle sample preparation method was developed, which is based on multistep solid phase extraction (SPE). A combination of chromatographic procedures and final concentration step are used to efficiently separate complex mixtures of compounds from natural sources. The procedure allows the generation of fractions of highly reduced complexity close to almost pure compounds. Depending on the complexity of the starting material and the purpose of the fractionated sample, the method allows any number of fractions, typically between 1 and 70, to be generated.

Using these fractions in automated biological assays makes test results much more reliable and reproducible. Furthermore, the quality of the provided samples allows them to be analysed without further treatment with methods like HPLC, TLC, NMR and MS. That enables rapid identification and structure elucidation of active compounds, as well as the recognition of known or already isolated metabolites.

### The new screening centre at the Hans-Knoell-Institute for national products research

*R. Thieriecke, K. Undisz and S. Grabley, Hans-Knoell-Institute, Jena, Germany*

The Hans-Knoll-Institute of Natural Products is an independent non-profit research institute in close partnership with the Friedrich-Schiller-University of Jena. Founded in 1992, the institute's purpose is the advancement of basic institute is organized in the divisions of Microbiology, Chemistry, Bioprocess Development, and Drug Discovery.

As a recent addition to the Hans-Knoll-Institute a screening centre has been established. The screening centre meets all the requirements for running efficient and successful drug discovery programmes in collaboration with industrial partners focusing on medical and agrochemical applications. The focus of our current research activities and ongoing projects are the realization of different screening programmes, the automation of new screening assays, the development of new sample generation procedures, and the bioprocess development. In addition to these applied aspects of drug discovery research the centre also carries out basic research towards the identification of new molecular targets.

### Homogeneous assay methods: the future of high throughput screening miniaturization

*Alfred J. Kolb, Packard BioScience Company, Meriden, CT, USA*

There are two major reasons why it is generally believed that homogeneous or non-separation assays will be required to meet future screening goals. These are time and miniaturization.

Even simple and relatively rapid steps like plate washing take on an entirely different time scale now that throughputs of 500 plates/day/target (50 000 samples) are possible. Semi-automated workstations are too labour intensive and tedious to be used with these throughputs. It is clear that a greater level of fully robotic automation will be required. Even though washing and filtration steps have been automated, homogeneous, mix and measure methods are much faster and far more practical for the required throughputs. While it doesn't seem like much, saving 1 min per plate with a 500 plate/day throughput adds up to a saving of 8 h a day. Homogeneous assays can save far more than this when one considers the time it takes to coat and wash plates or to separate by filtration or centrifugation. This far more efficient use of robotic workspace has an additional benefit of reducing the cost of equipment and facilities space.

Screening scientists cannot meet their throughput goals without miniaturization. While the 96-well plate is still the standard, the 384-well plate and the necessary equipment to process them are available. There are already significant advances being made in the development of 864-, 1536- and 3456-well plates. At some level of miniaturization, the methods commonly used in today's lab will no longer be practical. For example, the use of filtration as a separation step has been the cornerstone of receptor binding assays. However, there are currently no 384-well harvesters available so this method will probably be restricted to the 96-well format. Coating and washing for in-plate binding assays is now being done in 384-well plates, but it is not clear if this will be practical when the use of 864-well plates becomes routine. Homogeneous assays eliminate the need for any of these additional steps and the variability they add to the results.

Fortunately, there are now a variety of homogeneous (completely in solution) and non-separation (solid support attachments) assay technologies available that cover a wide range of targets such as biomolecular interactions (receptors, immunoassays, protein binding), enzymes and cellular assays. They are also available with radioisotopic, luminescent and fluorescent signals. The choice of assay technology has a significant impact on the level of miniaturization. As assay volumes are reduced from 100–200 µl (96-well) to 25–50 µl (384-well), 10–15µl (864-well) and lower, the signal being measured becomes critical. Because of half-life, specific activity and safety issues, there is a limit to how much signal can be obtained from a radioisotopic assay. Lower amounts of radioactivity can be quantitated, but the increase in counting time is contrary to the goal of high throughput screening. This will set a limit on miniaturization. In contrast, luminescent and fluorescent assays can achieve much higher signals for a greater degree of miniaturization. Examples were given of the miniaturization of isotopic, luminescent and fluorescent assays for biochemical and cell-based assays.

### Automated distribution of solid support

*Peter Kieselbach and Gregory L. Kirk, Pharmacopeia, Inc., Princeton, NJ, USA*

Commercial-scale combinatorial synthesis on solid support requires the distribution of large quantities of solid support resin, or beads, with stringent demands upon accuracy, cleanliness and throughput. Hundreds of thousands to millions of microscopic polymeric beads must be deposited into containers or microplate wells in a method which allows control over the number of beads deposited and which causes no damage or contamination.

Pharmacopeia and SCITEC (Wilmington, DE) have developed an automated system for distribution of solid support from bulk containers to 96-well microplates employing volumetric transfer of an isobuoyant suspension. Consumables are stored in automated carousels and transported through the system by a CRS robot. Volumetric delivery is accomplished by a Zymark RapidPlate pipeting workstation and the plates, containing the solid support beads, are washed by a Pharmacopeia-developed apparatus and dried in a vacuum drying oven.

The process challenges, architecture and performance of this automated system were presented and other methods of delivery discussed, including an automated bead-counting device for accurate delivery of an arbitrary number of beads, down to one bead per well.

### Bar codes and other long term identification methods for microtiter plates in high throughput screening

*Kevin K. Olsen, Wyeth-Ayerst Research, Pearl River, NY, USA*

Management of the large numbers of microplates used in a high throughput screening environment requires reliable marking methods. Typical conditions to which plates are subjected include solvents, rough handling and repeated freeze/thaw cycles from extreme cold room temperature and back again.

The problem is complicated by the nature of polypropylene which is commonly used for the initial compound dispensation. Polypropylene and its co-polymers are resistant to most solvents used in compound dissolution and distribution. Unfortunately, they are also resistant to inks and some label adhesives.

The labels can consist of one-dimensional bar codes for simple identification. These are highly reliable, easy to print and simple to read. More elaborate two-dimensional bar codes can both identify the plate and encode information about it. They are also highly reliable. Labels that are scratched, torn, or applied to irregular surfaces are still readable. The principle disadvantage is that they can only be read with specialized scanners.

One efficient marking method is polyolefin labels with acrylic based cold temperature adhesives. Another highly effective method is laser engraving. Wyeth Ayerst's Research Compound Bank uses this system for plates that are intended for long term storage. Other advanced identification methods include 'smart' tags that contain a memory chip that is activated by direct contact with a reader or by radio.

Whatever system is utilized, the ultimate goal must be reliability. Paying attention to details such as adhesives, label stock and bar code selection will result in years of trouble-free identification.

### A new fully automated mass spectrometer for confirmational analyses in drug development

*Karl-Heinz Maurer, Harald Gerken and Martin Maurer, AMD Intectra GmbH, Germany*

An AMD M-40 DF magnetic sector mass spectrometer of benchtop design is used in combination with a Zymate robot system to provide high throughput MS analyses in Direct Chemical Ionization (DCI)-mode. The system has been in operation for several months in the analytical laboratory of the drug research group of a large German pharmaceutical company. The major analytical target for this system is the automatic confirmation of molecular weights in combinatorial synthesis under high sample throughput confirmations with very high statistical probabilities and proves to be a very efficient and complementary method to ESI techniques. For a number of substance classes the DCI technique is the method of choice. The system functions were described, and the results presented.

### Automated Grignard addition to Weinreb amides: use of air-sensitive reagents on the Nautilus 2400

*Tracy Deegan, John A. Porco, Jr and Glynn S. Searl, Argonaut Technologies Inc., San Carlos, CA, USA*

The Nautilus 2400 synthesizer was used to automate the addition of Grignard reagents to Weinreb amide derivatives on solid support. The utilization of air and moisture-sensitive Grignard reagents demonstrated the utility of the Nautilus' inert fluid delivery system.

### Evaluation of matrix pooling process in lead identification: a case study

*Dennis J. Murphy, Peter J. Bouchard, Steve J. Link, Angela H. Gillespie, Wayne G. Carey and Thomas D. Y. Chung, DuPont Merck Pharmaceutical Company, Wilmington, DE, USA*

As chemical files increase in size there are pressures to increase the throughput of compounds tested while reducing cost of assays. One solution is to test pools of compounds. However, the increase in efficiencies are offset by the time and costs of retests and deconvolution, as well as the concern about the additive effects and masking effects of compounds. Matrix pooling is a method that functionally results in each compound being tested twice, each time with a different cohort of compounds, and theoretically eliminates the need to reassay each individual member of an active pool. We have screened a relatively large chemical files against a clinically relevant protease target both in pools and in singles, using a robust fully robotized assay. The data provide an additional case study of the necessary deconvolution processes and provide a comparison and analysis of the leads identified or missed by screening compounds in pools versus individually.

### Use of an automation compatible 24-well insert system for various cell based assays

*Amy Goldberger, Paul LaRocca, and Darwin Asa, Becton-Dickinson Labware, Bedford, MA, USA*

In recent years, the use of cell based assays for drug screening applications has increased tremendously. However, some cell based assays are difficult to use in a highly automated screening laboratory setting due to special handling conditions required for cells, or general format incompatibility of the assay platform with automation equipment. This is particularly true of cell based assays requiring the use of microporous membranes. Current microporous membrane systems for cell based assays utilize individual inserts which are not amenable to automation equipment. To overcome these barriers to using cell based assays in an automated screening setting, we have developed an automation compatible 24-well insert system for use in cell-based assays and tested the system in several cell-based applications.

Using a unique 24-well insert format, we have produced insert systems incorporating either a 1.3 or 8 μM PET membrane for different cell-based applications. The 1 μm PET system has been tested for its ability to support the differentiation of CACO-2 cells used for drug permeability studies. The 3 μM system has been tested for its ability to support neutrophil migration in response to chemotactic gradient. While the 8 μM system has been tested for its ability to support an assay for tumour cell invasion through an extracellular matrix protein layer. The data presented demonstrated the utility and performance of this system in several commonly used cell based assays.

### Automated parallel synthesis of olefins

*Joseph M. Salvino, Rhone Poulenc Rorer, Collegeville, PA, USA*

The Wittig and Horner–Ennomons condensation reactions are attractive and versatile methods to generate carbon–carbon bonds, producing olefins as products. The Wittig reaction has been utilized extensively on solid support, as well as the Horner–Emmons reaction. A process to produce α,β-unsaturated resin bond benzyl esters has been semi-automated, greatly facilitating the parallel solid phase synthesis of olefins for either biological testing or further synthetic elaboration. The automation protocol was described, including reagent weighing, robotics synthesis, evaporation, sample weighing and dissolution in preparation for high throughput screening.

### A versatile robotic system for the automated organic synthesis of compound libraries in the drug discovery process

*Will Kuijpers and Ton Jenneboer, NV Organon, OSS, The Netherlands; Eugy Van Gool, Labotec NV/SA, Teralfene, Belgium*

Within pharmaceutical companies there is a growing tendency to integrate automated synthesis systems into drug discovery research. With the aid of organic synthesizers, techniques such as solid-phase organic chemistry and combinatorial chemistry can be fully implemented in the drug discovery process. Several synthesizers have already been commercialized and are often integrated as workstations in semi-automated synthesis streets.

This paper described the development of a versatile robotic system which takes care of the entire synthetic process, including workup of the products (evaporation, extraction). The system is capable of performing both reactions on solid-phase and in solution.

The heart of the system is a new Zymark BenchMate-based synthesis block called LaMOSS (Labotec Modular Organic Synthesis System). This synthesis block contains 48 reactors with a reaction volume of 5 ml, organized in six units of eight reactors. Solvents, reagents and building blocks are added by a robotic arm, located in the centre of the units. LaMOSS further contains storage racks for tubes, building blocks and reagents, as well as a vessel to collect the resin in split-pool syntheses. Reactions are run under inert atmosphere (nitrogen). The reactors can be cooled or heated by air from a thermostration unit. Reflux conditions can be applied as well since the top side of the reactors can be cooled to 10°C. After completion of the synthesis and in case of a solid-phase synthesis after cleavage from the resin, the products are collected in glass test tubes. Subsequently, the tubes are placed in a shuttle and transferred to a Zymark XP robot which takes care of the workup of the samples. The latter process involves evaporation of solvents, and a liquid/liquid extraction to purify the products.

The robotic system has been operational since January 1997. A full description of the various elements was given, with the emphasis on the LaMOSS synthesis block. Results of a number of syntheses performed on the system were evaluated.

### Automated system for solid phase synthesis of combinatorial libraries

*Rajesh K. Maheshwari, Alex Berenshteyn, Bernard Neustadt, and Elizabeth M. Smith, Schering Plough Research Institute, Kenilworth, NJ, USA*

A robotic synthesizer has been developed to accelerate the generation of combinatorial libraries of molecules using solid-phase chemistry. This instrument has been in operation since January 1997. Libraries of single compounds and mixtures have been synthesized using this instrument, and HPLC and mass spectroscopic characterization of the products have shown very good purity.

The instrument completely automates the liquid handling operations of this process, and it is used for continuous batch-wise operation to provide regular output of samples for testing. It reliably handles the extensive data involved with synthesis of thousands of component molecules.

Several engineering challenges were overcome in developing a modular reaction block, which accommodates reaction vessels of 15 to 120 µl capacity. It can also conduct solid phase chemistry with resin-loaded microreactors. The top module of the reaction block is light enough to be removed from the system by the chemist with the reaction vessels maintained under inert atmosphere. Thus, it can be placed on external shakers, heaters, or chillers.

In addition to the modular reaction block, modular trays have been developed to hold the building blocks and reagents. The instrument is computer controlled, and the operator can manually intervene at any time in the reaction cycle and then restart the reaction in automatic mode.

A chemist-friendly programming language, SPOSL, has been developed through which the chemist can program the synthesis, making this instrument very versatile and flexible. Dispensing of building blocks and reagents can be specified by volume or as molar equivalents relative to the scale of the synthesis. Any existing multi-step method is readily modified to create a new method.

A special graphical synthesis planner has been developed in the software which allows the chemist to plan the synthesis graphically on a reaction vessel basis. For the purpose of compound registration, provision has been made for a link with external registration and enumeration software.

### Automated organic synthesis and process optimization

*Peter Hilberink, Frans Kaspersen and Eugy van Gool, N.V. Organon, The Netherlands and Labotec NV/SA, Teralfene, Belgium*

In 1994, a Zymark XP synthesis robot was installed at Organon for reaction optimization. This robot is able to start, control and finish organic reaction without supervision. Experimental design is used to efficiently run the experiments and to analyse the data generated by the robot.

During the last three years, some very useful results have been achieved, but we also identified some shortcomings of the system. The major one is that the system is not able to analyse the samples immediately. Because samples have to be analysed offline by GC or HPLC, they are stored for a period of time and are usually several hours old (up to 24 h) before analysis. During this time, the samples are undergoing degradation which influences the outcome of the experiments. In some cases, the results were highly questionable and the experiments had to be cancelled.

Because we were able to acquire a second Zymate II robot, we saw a chance to eliminate this shortcoming. The Zymate II receives the samples from the synthesis robot and performs sample preparation and HPLC injection. The design and some experiments were discussed.

### Automated dissolution using Zymate Assisted MultiDose (ZAM) and integrated HPLC system

*Scott Jennings, Bristol-Myers Squibb, New Brunswick, NJ, USA*

An automated dissolution system which integrates a MultiDose® Workstation, Zymate XP® robot and Hewlett Packard HPLC has been developed. This unique system allows us unattended dissolution analysis of the solid oral dosage form which needs chromatographic finish. ZAM allows us to transfer capsules and tablets to the MultiDose®, collect samples into HPLC vials and finally transfer the sample vials to the HPLC autosampler and back to the storage rack after the analysis. The unique features of this integrated system are that it performs HPLC analysis of the dissolution samples as they are collected and requires no manual transfer of the samples to the HPLC autosampler. It will also automate the removal of capsule sinkers at the end of a dissolution run thereby allowing us to test encapsulated products. This system will increase sample throughput by about 50% compared to a standard MultiDose®. A sizable productivity gain compared to the existing robot serial dissolution system is also expected.

Excel® is the control program that monitors the dissolution activity on the MultiDose and the progress of the HPLC analysis of the HP 1050 system. Two-way communication between Excel®, HP Chemstation, MultiDose® and System V® is achieved using Dynamic Date Exchange (DDE), serial RS232 communication and Zymark's Visual Basic-VBX®. In this presentation, the innovative hardware, software, and communication pathways were discussed. The presentation summarized the challenges encountered during the ruggedness testing and validation of such a complex system.

### Validation techniques for an automated dissolution system

*Daniel C. Hunneyman, Susan G. Kelley, Michael D. Stroz, Wyeth-Ayerst, Analytical Research & Development, Pearl River, NY, USA*

The Zymark MultiDose system has proven itself to be a valued analytical tool for dissolution laboratories. However, the MultiDose system validation and the transfer of validated manual methods to the MultiDose system have been a time-consuming obstacle for most laboratories.

An approach to simplifying MultiDose system validation and the cross-over validation of manual methods will be discussed. The presentation illustrated validation on the basis of proving equivalency to a manually operated USP apparatus 2. The proposed system validation requires three days to perform, and the proposed transfer of a validated manual method requires two days to perform.

### The effect of various operational parameters on the delivery of product through the valve of metered dose inhalers

*David Radspinner, Rhone-Poulenc Rorer, Collegeville, PA, USA*

An automated system has been designed, constructed, qualified, validated and implemented in our laboratories for the determination of dose-delivered-through-the-valve of various metered dose inhalers. This system is capable of wasting, collecting, weighing, and assaying by UV and HPLC. An experimental design was implemented to study the effect of a variety of parameters on the delivery of product through the valve. The levels of each parameter in the computer-aided experimental design were automatically input into the aerosol robotics system. The wasting and collection stations are capable of variable control of the shake speed and time, actuation duration, post-actuation delay, post-shake delay, and total number of actuations. An overview of the system design, the experimental design, and the results was given.

### Automating the analytical laboratory via the chemical analysis automation program

*Robert Hollen, Los Alamos National Laboratory, Los Alamos, NM, USA*

To address the need for standardization within the analytical chemistry laboratories of our nation, the CAA (chemical analysis automation) program within the US Department of Energy, Office of Science and Technology within the Robotic Technology Development Program is developing laboratory sample analysis systems that will automate the environmental chemical laboratory. The current laboratory automation paradigm consists of islands and automation that do not integrate into a systems architecture. Thus, today the chemist must perform most aspects of environmental analysis manually using instrumentation that generally cannot communicate with other devices in the laboratory.

CAA is working towards a standardized and modular approach to laboratory automation based upon the Standard Analysis Method (SAM) architecture. Each SAM system automates a complete chemical method. The building block of SAM is known as the Standard Laboratory Module™ (SLM™). The SLM, being either hardware or software, automates a sub-protocol of an analysis method and can operate stand-alone or as a unit within a SAM. The CAA concept allows the chemist to assemble an automated analysis system, from sample preparation through data interpretation, using standardized SLMs easily and without the worry of hardware or software incompatibility or the necessity of generating complicated control programs. A Task Sequence Controller (TSC) software program schedules and monitors the individual tasks to be performed by each SLM configured within a SAM. The chemist interfaces with the operation of the TSC through the Human Computer Interface, a logical, icon-driven graphical user interface. The CAA paradigm has successfully been applied in automating EPA SW-846 Methods 3541/3620/8081 for the analysis of PCBs in soil matrix utilizing commercially available equipment in tandem with SLMs constructed by CAA. The presentation included a short video showing the operation of the system.

™ Standard Laboratory Module and SLM are trademarks of SciBus Analytical Inc.

### A standard analysis method for the automated analysis of polychlorinated biphenyls (PCBs) in soils using the chemical analysis automated paradigm: validation and performance

*Charles Rzeszutko, C. Robert Johnson, and Matthew Monagle, Los Alamos National Laboratory, Los Alamos, NM, USA, and Leon N. Klatt, Oak Ridge National Lab, Oak Ridge, TN, USA*

The CAA programme, sponsored by the Robotics Technology Development Program within the Office of Science and Technology, US Department of Energy is developing a standardized modular automation strategy for chemical analysis. In this automation concept, analytical chemistry is performed with modular building blocks that correspond to individual elements of the steps in the analytical process. With a standardized set of behaviours and interactions of the blocks, these blocks can be assembled in a plug-and-play manner, into a complete analysis system. These building blocks, which are referred to as Standard Laboratory Modules™ (SLM™), interface to a host control system that orchestrates the entire analytical process, from sample preparation through data interpretation. The integrated system is called a Standard Analysis Method (SAM).

A SAM for the automated determination of PCBs in soils, assembled in a mobile laboratory, has undergone extensive testing and validation. The SAM consists of the following SLMs: a four-channel Soxhlet extraction, a high-volume concentrator, an extract clean-up, a gas chromatograph, a data-interpretation module, a robot, and a human-computer-interface. The SAM is configured to meet all the requirements specified in US EPA SW-846 Methods 3541/3620/8082 for the analysis of PCBs in soils. Once the input queue is filled, samples are automatically completed in intervals of 30 min. The PCB SAM was described along with the validation strategy. Validation and performance data from the testing protocols was discussed.

™ Standard Laboratory Module and SLM are trademarks of SciBus Analytical Inc.

### A total suspended solids automation system with a graphic user interface using spreadsheet data handling

*J. J. Zieman, P. L. Morabito, R. Tamilarasan, Dow Chemical Company, Midland, MI, USA; P. D. Hazelwood, Dow Chemical Company, Sarnia, Ontario, Canada and S. Sayers, Dow Chemical Company, Texas Environmental Analytical Laboratory, Freeport, TX, USA*

The Automation Team of the Dow Chemical Company has developed a Total Suspended Solids (TSS) automation system for environmental water sample analyses. The system uses Gooch crucibles with glass fibre filters to collect the solids from water samples. The crucibles are placed into a desiccation system with removable stainless steel racks. Sample delivery is performed using a peristaltic pump into a custom filtration station. Later, the crucible racks are manually placed into the drying or ashing oven. Dried TSS samples may be further processed by ashing to determine the Volatile Suspended Solids (VSS) content. This system can process a total of 60 samples per run without dilution or 30 samples with dilution before filtration.

A Microsoft® Visual Basic® graphical user interface was developed to incorporate point and click control of the system, including automated pump calibration. The graphical user interface incorporates all data entry and output into Microsoft Excel® spreadsheets. This automated data handling was essential and has allowed interrupted runs during off line drying or ashing of other batch samples.

### Microvolume screening of combinatorial libraries in high-density microtiter plates

*Jonathan J. Burbaum, Pharmacopeia, Inc., Cranbury, NJ, USA*

High-throughput screening (HTS) is a well-established method for the discovery of new pharmaceuticals. HTS is now becoming a focus for technological development because of significant advances in both combinatorial chemistry and genomics, which are increasing both the number of compounds and the number of targets. Pharmacopeia is pursuing a HTS-New Technologies (HTS-NT) system, which revolves around microlitre volume assays in 1536-well microtiter plates. This system has clear benefits not only to Pharmacopeia's drug discovery efforts, but also in many other arenas. Lower volume bioassays are widely advantageous, conserving both target reagents and compound collections. Reductions in volume also allow samples to be arrayed at higher densities to increase sample throughput.

This system presents a number of technological challenges to both biologist and engineers. A wide range of bioassays, including enzymatic, receptor binding, and cell-based assays need to be addressed in a microvolume format. Further, to carry out these assays in an efficient manner, improvements in liquid handling and detection, as well as the design and molding of new containers, will need to be implemented. The authors' approach toward meeting these challenges was discussed.

### Integration of the GENESIS with RoMa and ACTIVITYBASE for SAR screening

*G. R. Baker, C. R. Molloy, M. A. Snowden and P. Osbond, GlaxoWellcome R&D, Stevenage, UK*

The SAR Group from the Enzyme Pharmacology Department provides enzymological expertise to the medicinal chemistry programmes that run within the UK. The support of primary and selectivity assays across a number of therapeutic areas requires us to present, test and track compounds through over 40 assay protocols concurrently with minimal human resource. We have integrated AB (ACTIVITYBASE—ID Business Solutions) and two Tecan GENESIS with RoMa systems to achieve robotic flexibility with the workflow management and compound tracking that is required.

AB is a WINDOWS-based application that can be used to manage the many aspects of activity testing (i.e. compound tracking, data analysis and data storage). We have customized this application by developing modules that provide automated QA, internet publishing of SAR data, sample archive management and an interface to the Tecan GENESIS with RoMa called ROBOTSERVER. This VB interface polls the AB database for 'Test Request' which are compound lists that have a series of robotic 'Actions' assigned to them. As each batch of work is received, ROBOTSERVER constructs all of the relevant TLS (Tecan LOGIC Software—Kubus) INI and JobList files and initiates the RCS (RoMa Control Software—LaboSoft) scripts that control each GENESIS, from data retrieved from the AB ORACLE tables. This provides the group's scientist with an easy-to-use interface to the complex suite of robotic software ensuring that compound solvation and sample presentation is always consistent with the AB 'Protocols' selected to analyse the assay data, irrespective of the sample format. Current features include the ability to queue and prioritize the batches of work from multiple remote NT workstations and to manage a 100-plate custom hotel resource on each of the GENESIS systems. The development of the system was discussed, together with future plans, including the integration of a new scheduling program into this system.

### Use of the Zymark RapidPlate-96 to perform pipet-in-a-tip assays for high throughput screening

*Crafford Harris, Ruth Brosius, and Mary Jo Wildey, The R. W. Johnson PRI, Raritan, NJ, USA and Andy Zaayenga, Zymark Corporation, Hopkinton, MA, USA*

In Drug Discovery at the R. W. Johnson Pharmaceutical Research Institue, high throughput screening is a dynamic programme whose objective is to maximize the effectiveness and efficiency of the drug discovery effort, resulting in the identification of new and novel pharmacophores. To that end, new concepts and techniques in reagent handling are always being investigated. One of the tools currently used in our programme is the 96-channel pipettor which is integrated into a custom Zymark robotic system. Two time consuming steps in an HTS screen are moving tip racks to and from the work surface and accessing assay plates multiple times to add multiple reagents. One obvious way to increase screening efficiency would be to limit the number of these types of robotic arm movements by utilizing the 'pipet-in-a-tip' concept. In this technique, all assay reagents are aspirated into a single pipet tip, with each reagent separated by an air gap to reduce preincubation problems. When one uses a 96-channel pipettor to accomplish this task, the throughput is enhanced even more. We have evaluated the Zymark RapidPlate-96 on our custom robotic system for this task. In this presentation the authors outlined this pipetting strategy and provided data on how this technique has worked.

### Development of an automated, high throughput reporter gene assay

*Richard K. Harrison, Research Fellow, New Lead Generation Department, Rhone-Poulenc Rorer, Collegeville, PA, USA*

In the last few years the pharmaceutical industry has witnessed an increase in the number of high-throughput cell-based assays. Due primarily to advances in molecular biology, simple, robust reporter gene assays capable of monitoring and reporting on transcription events is now commonplace. However, as with most high throughput screens, many problems are met when taking an assay from the manual, one-plate ('bench') scale to an automated, multi-plate scale. This talk focused on the implementation and validation of several automated high throughput reporter gene assays, highlighting the advantages and disadvantages of each, and offered solutions to several of the common problems associated with implementation of these assays.

### Integration and organization of an HTS Group within drug discovery

*Michael Snider, Berlex Bioscience, Richmond, CA, USA*

Empirical screening has become a routine technology in early drug discovery research in the pharmaceutical and biotechnology industry. While the specifics of how screening is accomplished between organizations (robotics versus workstations; pooling versus single compounds), there are still many similarities. However, there are two factors which are often underestimated in terms of importance for success: the organization of the screening group within Discovery Research, and the nature of the specific biological targets which are chosen for screening efforts. Additionally, the human element of the automated screening operation was discussed, as well as the impact of genomics on Discovery Research dynamics.

### The integration of parallel combinatorial unit chemical synthesis and multiplexed, high throughput bioassays to accelerate drug discovery

*Stewart D. Chipman, Adrian Sheldon, John Petracca, David S. Casebier and Joseph C. Hogan, ArQule, Inc., Medford, MA, USA*

Scientists engaged in drug discovery have benefited greatly by the application of laboratory/industrial-scale automation to both biological testing to enable high-throughput bioassays, as well as chemistry to enable parallel combinatorial chemical synthesis. The authors described an approach to the integration of parallel combinatorial unit synthesis and high-throughput bioassays that maximally accelerates the discovery of lead structures from ArQule's 200 000 + chemical compound Mapping™ array against multiple biological targets.

ArQule has developed and reduced to practice the automated, parallel, solution-phase synthesis of large ($10^3$ to $10^4$) compound sets, or arrays, of small organic molecules. Each array contains a unique backbone chemistry with diverse pharmacophoric side groups attached. These arrays are created by combinatorially reacting a serial of functionally identical but structurally diverse building blocks to create a single compound which is chemically analysed by HPLC and mass spectrometry. The compounds are logically arranged in spatially addressable 96-well microtitre plates with a single compound per well. This format yields an information rich array where chemical structure, mass, and other properties are stored in a chemical database that is searchable via a unique identifier. These arrays have been assembled into a larger set of 200 000 + compounds referred to the ArQule Mapping™ array.

Several strategies are typically employed to manage the biological testing of a large chemical compound sets (i.e. ArQule's Mapping™ array) against multiple biological targets. Single compound per bioassay per well is the most direct. The advantages are that no deconvolution is required and minimal potential for 'masking' of bioactivity exists. Single compound per bioassay fits particularly well with the information rich nature of ArQule's Mapping™ array; essentially the entire primary bioassay provides extensive SAR data, the negative bioassay data also adding value for the subsequent lead optimization activities. However, the cost of this approach is significantly higher than the alternative strategy of compound pooling. Pooling of between 3–10 compounds/bioassay has been utilized to quickly and efficiently assay large compound sets (1,2). The primary disadvantage is the need for subsequent deconvolution of positive readouts, the potential for masking of one compound's activity by others and, specifically for ArQule's Mapping™ array the information content of the compound set is partially lost.

In order to efficiently bioassay large-compound arrays while preserving the integrity of the SAR information content, we have found that multiple, parallel, high-throughput bioassays are an attractive alternative. The authors looked at several case examples of multiplexed bioassays with ArQule's Mapping™ array and discussed issues related to data management, prevention of readout crosstalk and multiplexing of primary and secondary assays.

### Integrating lab automation into the drug discovery process: current success and future directions

*Richard H. Griffey, L. L. Cummins, S. Owens, H. Sasmor, R. Bergert, V. Mohan, E. Swayze, J. Wyatt, S. Freier, D. Ecker, and P. D. Cook, Isis Pharmaceuticals, Carlsbad, CA, USA*

Lab automation has broad impact in all aspects of the drug discovery process and creates new challenges for information management. The greatest impact has been realized not for repetitive tasks, but in areas where human skills can be leveraged effectively. The synthesis and purification of lead compounds can be automated using solid-phase multiwell synthesizers or by automated purification of complex mixtures prepared by chemists. Analysis of compound identity is performed routinely with automated mass spectrometric or NMR services.

Drugs are screened against a variety of biological targets using high-throughput assay systems. Pharmacokinetic and metabolic profiles can be generated using automated extraction and analysis methods. While automation has improved productivity in all of these areas, the available information management tools are inadequate to store and integrate the quantity of data. In the future, the time for discovery of drug development candidates will be shortened dramatically, as genomic information is used to identify protein and nucleic acid targets, and lead compounds are prepared, purified, and screened in the automated lab.

### Chasing the rate limiting step

*Mark Jones, Astra Charnwood, Loughborough, UK*

Bringing new drugs to the market is an extremely slow and expensive process with a modest success rate. In recognition of this fact, the theme for the 1990s has been 'more for less and faster'. The introduction of automated systems has played a major role in our attempt to achieve this goal and it has proved to be an extremely liberating experience.

It has provided us with the opportunity to question the things we do and why we do them to focus attention on the rate limiting steps in the research process. We have seldom leapt to automate our existing work practices, instead we have explored how new technology can help us to develop new work practices resulting in significant improvements in productivity and quality.

It has been our experience, that by turning a 'rate limiting step' in to a highly efficient machine merely serves to move the problem to another part of the research process, consequently we find ourselves on a seemingly endless journey of 'chasing the rate limiting step'.

### Technology transfer of automated methods—our early struggles

*William B. Tucker, Bristol-Myers Squibb Company, Evansville, IN, USA*

In our new global economy, analytical transfer has become increasingly important to the industry and regulatory agencies. It has become a special concern for the pharmaceutical industry and the FDA. Analytical technology transfer for automated methodologies is the next logical step in this process; however, it does present some unique issues. As with all technology transfer, it requires a balance of four key ingredients:

- A rugged method.
- Comparable equipment.
- Proper training.
- Mutual desire for success.

Using examples from recent experiences, the importance of each of these ingredients, and the lessons learned were discussed.

### Automated method validation and transfer in a QC environment

*Muhammad Alburakeh, Marc Rosenberg, Limin Zhang, Jon Sadowitz and Donald Nguyen, Barr Laboratories, Pomona, NY, USA*

Process validation requires extensive analysis to assure the uniformity of the blend, final dosage form and drug release profiles at different stages of the manufacturing operation. Use of robotics to automate the testing procedures increases productivity and shortens the turn around time for the validation results and ultimately approval and marketing of a drug product. For the first time we are able to successfully automate the complete testing of a drug product to support process validation. The dissolution, blend uniformity, content uniformity and assay testing of a drug product was automated using a Batch Dissolution System and a TPW II workstation. The key to automating the testing procedures is the validation and transfer of the methods to the QC laboratory. This presentation covered the steps that are necessary for the implementation of automated methods in a QC environment.

### Analytical testing alternatives, investment approaches

*Brian Hausner, Pharmalytic, Inc./Micron Technologies, Exton, PA, USA*

Today's pharmaceutical companies are undergoing radical changes in the way they operate. This is necessitated by greater government scrutiny, increased activity of HMOs and other 'buying co-ops' and the evolution of healthy lifestyles. All factors contribute to increasing attention to cost but also on eventual new products. Traditional responses have been a recent wave of mergers and moving operations to new tax havens that create implications on a global basis, especially operationally. Finally, numerous firms are focusing on core competency, as they define this to mean usually drug discovery/development and market/distribution.

Laboratory personnel need to understand their companies' strategies/objectives. What are the core competencies as well as strategy drivers? These drivers could be capacity or technology related, or simply to shorten the product development cycle. This can be accomplished by increasing risk by managing multiple projects or steps within a project simultaneously. The use of fianancial models can help support decisions based on 'intuitive' knowledge. The types of models can be as simple as cash flow analysis to Net Present Value and even EVA (Economic Value Analysis). Financial models can help balance the level of acceptable risk and goals/strategies.

How to assign value to key drivers was reviewed using strategic decision making programs. This information was then used across the numerous investment alternatives faced by lab personnel, using some of the previously discussed drivers. The investment alternatives to be considered are laboratory automation, temporary staffing, use of contract analytical labs and finally internal investment; i.e. headcount increase.

### Fully automated SPE-LC-MS

*R. A. Biddlecombe and S. Pleasance, Glaxo Wellcome Ware, Hertfordshire, UK*

The authors have previously reported on the application of the MicroLute system of solid phase extraction (SPE) in the 96-well format for high throughput bioanalysis using LC-MS-MS. The original system used a customized vacuum manifold located on the deck of a Packard MultiPROBE robotic sample processor. The MultiPROBE performed all the liquid handling steps, but the operator is required to manually place the collecting plate in the vacuum manifold prior to the elution step. In order to maximize the advantages of this approach and overcome some of the limitations, a fully automated standalone system has been developed. The system consists of a Zymate XP robot, cooled storage carousel which acts as both a warehouse for all the labware and as a refrigerated storage for the extracts, and custom built SPE station. The SPE station incorporate a vacuum manifold, reagent addition strip (RAS) for rapid reagent addition a row at a time and solvent switching valve which allows up to nine different reagents to be used on the system. This station allows all the vacuum steps, conditioning, washing and elution steps to be performed much faster and the MultiPROBE is used simply to dilute samples, add internal standard and transfer samples from tubes to plates. The plates containing the extracts are returned to the cooled storage carousel to await analysis. The acceptance criteria was to perform a minimum of four consecutive unattended runs (384 samples) and to be capable of handling all the current available labware (Microlute and Empore blocks).

Data were presented from several LC-MS-MS methods using this approach and the phased development of the system into a fully automated SPE-LC-MS-MS system that can directly feed a PE-SCIEX API-300 mass spectrometer and is capable of round the clock operation was detailed.

### Use of automated solid phase extraction for a validated assay for prostaglandins in human urine using gas chromatography electron capture negative chemical ionization tandem mass spectrometry (GC ECNCI MS/MS)

*Clark V. Williard, Ajai K. Chaudhary, Karen M. Clements, Robert J. White, Thomas D. Oglesby, and Paul A. Taylor, Taylor Technology, Inc., Princeton, NJ, USA*

Prostaglandins (PGs) play an important role in a variety of diverse biochemical processes. A large number of endogenous PG analogues have been characterized with small structural variations. They are generally present in extremely low concentrations. GC/MS assays have been developed for PGs, but suffer from the presence of coeluting impurities which makes the measurement difficult and irreproducible at lower concentrations. We have modified the existing assays to develop a highly sensitive and robust assay for $PGE_2$ (an indicator of kidney PG metabolism) and 6-keto-$PGF_{1\alpha}$ (an indicator of prostacyclin production), from human urine. The lower limit of quantitation for both the analytes is 10 pg/ml using 1 ml human urine. The method involves elaborate extraction and derivatization steps. A Zymark-RapidTrace SPE Workstation was used to automate the extraction of analytes from human urine (in reversed phase SPE mode) as well as to purify the pentafluorobenzyle derivatives from the reaction mixture (in normal phase SPE mode). In addition, use of tandem mass spectrometry provides an additional degree of specificity to the assay. The assay has been validated following GLP guide-lines and is being used to assess endogenous PGs production.

### High throughput method development with microtiter solid phase extraction plates for bioanalytical mass spectrometry

*John Janiszewski, Monica Swyden and Hassan Fouda, Pfizer, Inc., Central Research Division, Groton, CT, USA*

A semi-automated protocol for solid phase extraction (SPE) methods development using microtiter, 96-well, SPE plates has been developed. The technique utilizes method development plates containing four sorbents in groups of three across the columns of the plate. The plates are processed using a programmable 96-well pipettor, the Quadra 96 (TOMTEC, Hamden, CT), and laid out in an array such that each analyte is screened across four sorbents an four eluents in duplicate. Fast-gradient (3 min/sample) LC/MS conditions are used to determine recovery.

Multiple analytes (3–5) are spiked into plasma and a 100–200 ul aliquot of the fortified plasma is transferred to the appropriate wells in a deep-well (1.2 ml) polypropylene block. The plasma is diluted 1:1 with 1% phosphoric acid prior to extraction. This sample block is then transferred to the desk of the Quadra 96 for SPE processing. The conditioning, load and wash steps have been standardized and the elution volume is 100 ul. The eluent (e.g. methanol, acetonitrile) is collected in a 96-well polypropylene plate and combined with 50 ul of water prior to LC/MS analysis. This technique uses a third (four columns) of the SPE plate for a given set of analytes. In its present application three method development experiments per plate are possible. The combined setup, LC/MS analysis, and data reduction time for a single experiment is approximately 4 h.

The optimal set of extraction conditions, yielding best recovery, allows rapid method development for pharmacokinetic analysis in support of drug discovery. Refinement of the SPE conditions are sometimes needed to extend the limit of quantitation. We have tested numerous (>100) compounds using this approach, 90% had recovery of 70% or better and 95% had recovery better than 50% from plasma. Experience with method development technique using both the 3M Empore and Porvair style microtiter solid phase extraction plates was described.

### High throughput sample preparation for LC-MS/MS bioanalysis using 96-well SPE plate and robot

*H. Simpson, D. Buhrman, R. Burton, J. Newton and D. Wu, Sanofil Pharmaceutical, Inc., Malvern, PA, USA*

The 96-well solid phase extraction (SPE) plate offers a rapid preparation approach for the analysis of drugs and metabolites in biological matrices, based on simultaneous extraction of 96 samples. In addition, the SPE plate operation can be automated by robots, further increasing productivity while reducing labour cost. In this study, a thin disk (membrane) containing chromatographic packing material immobilized within polytetrafluoroethylene carrier (Empore™ disk) has been incorporated into the 96-well plate as the SPE sorbent. The small bed volume in the disk plate resulted in small elution volume (75–200 pl), allowing direct injection of the elution fraction to LC-MS/MS at the end of the sample preparation; and significantly improved sample-to-sample reproducibility due to reduced channelling effect on the disk. The performance of the disk plate was characterized in this presentation by compounds of wide range of basicity to represent pharmaceutical agents. Optimal conditions were evaluated based on the volume, flow rate, vacuum control, mass recovery, potential clogging, use of acid to elute basic compounds, etc.; the results were presented. The automation of the disk SPE plate operation was accomplished by a modified MultiPROBE™ 204 DT robot. A computer controlled solenoid valve was incorporated into the robot to control the flow rate of solvents with varying viscosity. A software procedure, 'Check Well', was developed to search for clogged wells based on liquid sensing, to avoid overfilling of the wells and potential contamination of the adjacent wells. The sample preparation using 96-well disk plate by a robot adds enormous value to a bioanalytical lab. The data obtained from several Sanofi compounds (for example, SR 46559, SR 31747 and SR 46349) were presented to demonstrate the utility of the technology. All these compounds were validated to the minimum quantifiable level of 0.2 ng/ml or lower. The typical accuracy and precision were $<5\%$ either within or between runs. The validated methods have been successfully applied to clinical sample analysis. The throughput and assay performance were reported.

### Towards comprehensive combinatorial chemistry information management

*J. Christopher Phelan and U. Josh Patel, Arris Pharmaceutical, South San Francisco, CA, USA*

Combinatorial chemistry creates a need for new methods of data handling that allow tracking of synthetic procedures, precursors, product structures, and analytical and assay data for thousands of compounds per months. The throughput needed requires that human intervention in the data handling be minimized. Maintaining accuracy of the data with respect to physical reality requires that the data path be tightly correlated with the physical production of actual combinatorial libraries. The authors described progress in addressing these issues in a context where chemistry development, production, and analysis of combinatorial libraries were performed by separate workgroups. Data structures designed to represent the actual steps in a combinatorial synthesis including reactions, protocols, precursor sets, and physical layouts were present. Design and implementation of software on multiple platforms to handle these data structures concurrently with the actual synthesis of combinatorial libraries were discussed. Goals included automated generation of robot operating instructions from synthesis specifications, automated generation of product databases including tracking information from actual synthetic procedures, subsequent incorporation of analysis and bioassay data into these databases, and quality management issues for the various stages of this process.

### An integrated system of data generation, analysis, and management for use in an HTS environment

*Mark J. Suto and Laura J. Schove, Signal Pharmaceuticals, Inc., San Diego, CA, USA*

A key component to the success of any drug discovery programme is the integration of HTS and combinatorial chemistry with database management and molecular diversity systems to yield an efficient and cost-effective method of organizing chemical information and biological data. At Signal, we initially loaded the HTS data into familiar applications such as Microsoft Excel, HyperCard, and local ISIS/Base databases, but their capacities were quickly overwhelmed by the immense amounts of data. We immediately saw the need for an integrated, efficient HTS information management system. Using MDL's SCREEN and ISIS/Host, we linked Oracle-based biological data and corporate identifiers with structural and catalogue information for data storage of biological and management. In addition, we integrated into this system Tripos's Chemical Diversity management suite of modelling software. The integration of HTS and combinatorial chemistry for rapid data generation, molecular diversity techniques for data analysis and evaluation of samples to be tested, and database management systems for the integration and storage of biological and structural data was achieved. This approach provides Signal with a comprehensive, efficient, and cost-effective programme for the discovery of novel therapeutics. In the future our biggest challenge information management will be in how we administer and optimize data as we work to validate leads and develop them into drugs. The success of many companies in facing this task will depend not only upon solid scientific expertise, but on a willingness to adapt and change both traditional views of drug discovery and methods for communicating and handling results.

### A robot's story: from take down and move to operational in two weeks—fact or fiction?

*Allison Manners and Howard Balter, Eli Lilly Canada Inc., Scarborough, Ontario, Canada*

Today's laboratories are focusing more and more on automation to either increase their analytical capabilities or to utilize scientists' resources elsewhere in the testing facility. A substantial amount of time and effort is needed to establish automated programmes and to validate the operation of the robotic systems. Therefore, once a robotic system is fully functional and validated, the last imaginable situation would be to dismantle the robot and move it to a new location. However, in today's changing environment in the pharmaceutical industry, these types of events are a regular occurrence. Moving an automated system should not be something to fear.

In late August 1996, the Lilly Analytical Research Laboratory now located in Scarborough, Ontario, Canada, moved locations. Prior to the move, two automated systems were fully validated and in routine use running potency, related substance, and preservative assays for a variety of pharmaceutical dosage forms. By mid-October of the same year the systems were once again fully validated and operational in the new facility.

The basis of this presentation was the steps implemented to make the transition between sites as seamless as possible and to share the learning acquired during the move. The need for appropriate planning, organization, and resources was discussed, as well as some unique short-cuts.

### Enhancement of sample preparation through new technology

*Jay Desai, Ortho-McNeil Pharmaceutical Corporation, Raritan, NJ, USA*

By enhancing the existing robot capabilities with new technology the Quality Assurance Analytical Laboratory was able to achieve its new cycle time initiatives for sample throughput. Initially, the mission of the QA Lab was to reduce cycle time for Ortho Novum content uniformity testing. With this primary goal in mind, we looked at the robot preparation time and testing procedure. We knew that we would have to reduce our sample preparation time. A decision was made to upgrade the robot by modifying its configuration. The following changes were made:

- A new over the balance reagent addition and tablet addition station.
- A pipetting station.
- An EasyFill vial filling station.
- The method was scheduled using Zymark's System Productivity Software package.

With these upgrades the cycle time was reduced significantly.

### Managing laboratory automation: a case study into the automation of a high volume dissolution laboratory

*Patrick Drumm, Novartis, Summit, NJ, USA*

In February 1996 as a result of both increased laboratory demands and our inability to add staff, we were forced to look to automation to fulfil our future objectives. A quick survey confirmed that our centralized dissolution laboratory was in fact the best place to start. A team was formed composed of both automation and laboratory chemists. They were charged with presenting to management a defensible automation plan and then with the more difficult task of implementation and validation.

This paper discussed the key elements of the laboratory automation process. In particular, it focused on:

- A team-based approach: It is essential that the right people be brought together, with the guidance of management, to solve these difficult problems. Solutions developed from within, although not always possible, should be the goal. These internal solutions are the most likely to succeed in the long run, since they have the full commitment of the lab group.
- A sound automation plan: This important area includes tools such as process flow diagrams and cost benefits analysis. In addition, considerable research is necessary to form a complete list of alternative solutions.
- Carefully assigned objectives, responsibilities and accountabilities: An individual 'champion' should be selected for each automation project however minor. These individuals should be given adequate resources (time) to support their project.
- Equivalence to the existing validated method: This is the essence of validation and represents the single most powerful argument for acceptance of the automated method.

### Data manufacturing: transition to an industrial mindset for laboratory automation

*James M. Gill II, Glaxo Wellcome, Inc., RTP, NC, USA*

The introduction of high throughput screening as a tool for discovery pharmaceutically useful compounds has led research to shift from work practices grounded in the craftsmanship of science to processes based in manufacturing. This change has occurred for two reasons. First, industrial scale automation is required to handle the increase in the volume of assays required by HTS. Second, and perhaps most important, just as industry must minimize the cost of each widget produced, the success of HTS depends on optimizing the value of each data point produced. The starting point for optimizing work lies in correctly capturing work practices in an algorithmic or rule based form. Once practices have been captured they can be quantitatively analysed and optimized. The industrial revolution in lab automation has also broadened the scope of automation requiring analysis of the cost of processes across many independent systems. Our group is using techniques borrowed from industry such as a statistical process control, automation quality control, statistical experimental design and implementing low function but highly parallel workstations. The use of those techniques in the context of the move from 94 to 384 well assays was discussed.

### Adapting PCS to non-Zymate architectures

*Dinesh S. Thakur, Vincent W. Chen and Kirk J. Leister, Bristol-Myers Squibb Company, Syracuse, NY, USA*

Bristol-Myers Squibb has developed and implemented a microplate assay system that utilizes extension components for a modified version of the Productivity and Communications Software (PCS from Zymark Corporation) for scheduling and coordinating peripheral hardware. This System hardware includes a Sagain ORCA robot, a RapidPlate-96 Workstation, two Tecan Genesis

Liquid Handling Workstations, a Zypettor, two Zymate XP robots (one serving as the primary robot for PCS and the other a part of an on-line reagent preparation station), and a custom GUI front-end.

The ORCA is used as a peripheral component and is controlled by an extension component to PCS using DDE as the interprocess communication mechanism. The RapidPlate-96 Workstation is controlled as a peripheral component via NetDDE in order to be used as a stand-alone peripheral. The two Tecan Genesis workstations are controlled by an extension component that coordinates the tasks performed by these workstations. The Zypettor and the two XP robots are controlled via the System V controller by the modified PCS. This system uses a front-end with custom interfaces to collect information and create a dynamic run-time schedule.

Typically, PCS supports Zymate architecture where one robot coordinates all peripheral hardware and additional robots are connected via local controllers to the main controller. Laboratory Unit Operations (LUOs) performed by the robots connected via local controllers are addressed as batch operations by PCS. This system uses the ORCA to get and put labware from the Tecan Genesis Workstations and the RapidPlate-96 Workstation, tasks typically not addressed as batch operations. The architecture implemented for the resolution of run-time variables collected by the custom front-end, the coordination of ORCA tasks, Tecan Genesis liquid handling workstations and the RapidPlate-96 workstation with the extension components were discussed in this presentation.

### Specification testing robot—an automated analytical laboratory

*Perry Hailey, Pfizer, Sandwich, Kent, UK*

While laboratory automation has grown in complexity, there are significant technical and business benefits in employing a modular approach when selecting instruments for automated systems. Rather than use a single complex instrument to perform a variety of tasks, a modular approach separates the various tasks among a number of specialized robotic units. Each of these instruments is designed for a specific task, often built by multiple vendors, and is capable of independent operation from the other modular units within the overall robotic system. This modular approach has significant advantages in that each unit can be operated independently by analysts, the failure of one modular component does not effect the overall performance of the automation design and also allows for opportunities to develop and modify the system as requirements change. Associated with the modular approach to instrument design is a modular approach to software. Software such as LabView or Visual Basic supports the concept of modularization, especially Labview which allows the construction of small modular code. It is important when developing to select a stable and secure operating system and Windows NT satisfies these criteria.

The overall concept of the specification testing robot is to deliver a robotic system capable of performing the majority of the testing requirements contained within a typical specification for the testing of drug product batches into an integrate system. The modular approach seeks to break the overall testing regime into component parts and this was described. The importance that IT plays in delivering the system was also discussed.

### Development of workstations for filtration and purification of samples prepared by automated synthesis

*David A. Rudge and Mandy L. Crowther, Zeneca Pharmaceuticals Ltd, Macclesfield, Cheshire, UK*

Automated organic synthetic chemistry has been undertaken in the Robotics Laboratory at Zeneca Pharmaceuticals since 1992 using Zymate™ XP Laboratory Robots. Isolation of products by a variety of procedures for the test in biological screens from a wide range of chemistries has been essential. The option to filter solid materials, from reaction mixtures and, at times to purify the products produced has required the manufacture of new modules to accommodate these demands.

Constant development has been undertaken in the area of filtration due to the increased volumes utilized on the synthesis robots. Systems have now been designed to allow off- and on-line chromatographic purification of samples up to 1 g using Mega Bondelut™ Chromatography cartridges.

The authors' experiences in developing these two procedures for product isolation were described.

### Development of an automated chromatography system

*Gary C. Walter, Clare Elliott and Karen F. Higgins, Zeneca Agrochemicals, Bracknell, Berks, UK*

With the development of efficient systems for robotic syntheses, sample purification has become a bottleneck in the process of production of new chemicals. This paper described the development of an automated chromatography system that is capable of processing 40 samples in parallel. The system can provide gradient elution and has the ability to change the gradient profile. Ten fractions are collected for each sample and are then analysed by thin layer chromatography using a Gilson liquid handling robot. A description was given of the way in which the new system improves productivity and turnaround time in comparison with existing equipment.

### The future of compound distribution at Glaxo Wellcome

*Brenda Johnson Ray, Glaxo Wellcome Inc., Research Triangle Park, NC, USA*

Compound distribution for discovery research has evolved so much over the last several years that a set of standard operations becomes obsolete almost before it is implemented. The ever changing requirements for screening have left the door open for new sample formats, container types, automation, storage, barcoding, inventory tracking and even the amount of compound required for testing. When will these rapidly changing requirements settle down, evolving to a set of best practices for compound distribution and screening? The answer doesn't seem to be in the foreseeable future. This presentation outlined how the Discovery Compound Distribution group at Glaxo Wellcome has advanced its sample handling practices so far, and continues to plan for the uncertainties of the future.

### Documentation and validation activities in the development of a robot system for the testing for sterility

*Elisabeth Averdam and Klaus Haberer, Hoechst Marion Roussel, Frankfurt, Germany*

A particularly important function of the Microbiology Department of Quality Operations is to minimize calculable risks and ensure that test parameters are as constant as possible. In the test for sterility, contamination by means of, for example, manual intervention must be prevented. The automation of this test by the introduction of robots could greatly contribute to the aforementioned goals. The suitability of such robots must be tested and documented with respect to their subsequent application in the strictly regulated pharmaceutical field. The robot must comply with a large variety of legal guidelines and technical standards, 'in-house' standards laid down by, for example, engineering or quality assurance departments, as well as technical recommendations and guidelines laid down by scientific societies.

To facilitate the validation of the complex computerized system in compliance with the various requirements as mentioned above, detailed validation protocols were purchased with the robot as a framework for the documentation. Only by cooperating closely with the companies involved was it possible to successfully bring about the necessary new developments in the field of technical accessories. A change control procedure was used during assembly of the robot as the PyTechnology system originally used had to be changed to a linear-axis system. The milestones of the project include design qualification, the works approval check lists, installation qualification, operation qualification and performance qualification which are documented with corresponding protocols.

This paper dealt with the structure of the formal documents integrated in the overall documentation together with the concept, the manufacturer's specifications and validation plan. Requirements and first experiences up to and including operation qualification were presented.

### A fully automated robotic solution for QC HPLC: A real life story about justification, implementation and operation

*Edward J. Fennessy, Jr, Pfizer Inc., Brooklyn, NY, USA*

The Brooklyn NY site of Pfizer Inc. is integral for the scale-up and manufacturing of newly developed products to supply pharmaceutical samples to the sales force as soon as the produce receives approval from the FDA. Several manufacturing steps are required to provide modern pharmaceuticals to market, and hundreds of analytical methods are employed to ensure product quality. Quality control is a partner with manufacturing and research, and is responsible for gathering data to support the manufacturing, packaging and stability of products. The preferred method for assay and uniformity characterization of the manufacturing process utilizes high performance liquid chromatography (HPLC). Representative samples are received from the various manufacturing steps including raw materials, mixed blends, tablet cores, and finished goods.

The Brooklyn Quality Control unit tests approximately 80 000 samples per year using HPLC. Testing usually involves four steps: sample preparation, chemical analysis, result generation, and data review and approval. Over the years, instrumentation has been improved for chemical analysis, and data acquisition systems have become proficient in processing data for result generation. Sample preparation is usually the most time-consuming testing component, and due to the repetitive nature, can lead to testing errors. Robotic applications are currently available to assist laboratories to automate sample weighing, disintegration and extraction, dilution, filtration, injection onto the HPLC system, and result generation. Automation's individual tasks are seldom fast, but when concatenated, can provide more efficient, accurate, and precise data. Additionally, environmental benefits can be realized due to reduced solvent consumption and emissions, and the skilled analyst can be freed of tedious work to perform more complex tasks.

The justification, implementation and operation of a fully automated robotic system were discussed. The criteria for selecting automated applications, appropriate steps to validate the robotic system and analytical methods, and potential advantages and disadvantages were presented.

### Image capturing in high throughput systems and global fingerprint monitoring™ automated visual Go/No-Go decisions

*Richard P. Gill and Richard H. Selinfreund, Lion Laboratories, Old Saybrook, CT, USA*

The standard high throughput system (HTS) generated reams of numerical data that must be processed and eventually interpreted by operators. HTS operations require complex initial start-up calibrations, material pre-qualification, on-line process control, go/no-go decisions and final process analysis. Using numerical analysis to control HTS significantly slows down operations. In fact, numerical analysis control points can cause incorrect go/no-go decisions under HTS speeds. Utilizing the technique of Real Time Image Capturing allows the HTS controller and operator to make significantly more accurate go/no-go decisions.

Real Time Enhanced Image Capturing is possible using computerized digital imaging systems built into the microplate scanner units. The digital image is then automatically analysed by software that processes images and numerical data. Standard Images can be stored in an optical database that provides the baseline for calibration and process control. Standard optical images provide optical fingerprints. HTS standard images can be rapidly compared to on-line images to make improved go/no-go decisions. Furthermore, final process analysis of numerical data can be coverted to optical images using Global FinerPrint Monitoring Software$^{TM}$. Ultimately, optical image analysis makes the task of operating and analysing HTS results simple and accurate. The paper described HTS process control using optical imaging and a process to automatically convert numerical data to useful optical images.

### Diogenes at Delphi: evaluating software for high throughput screening

*John Morin, Jaweed Ashraf, Fred Immermann, James LaRocque, Fernando Ramirez, Linda Heydt, T. C. Ramaraj, Imad Mansour, Mariya Gazumyan, Michael Greenstein, Ian Fore, Dave Vanderbrooke, Sheryl Jeffas, Gary Kuehnlenz, Diana Adams, Ronald McCaully, John Babiak, Wyeth-Ayerst Research, Pearl River, NY, USA*

Timely data management is a significant challenge for most high throughput screening groups. Our data management procedures have evolved through many stages over the years. We have progressed from laboratory notebooks and other paper trails to ad hoc use of Microsoft's EXCEL and Access and through a short succession of customer systems developed by our corporate research computer departments. Our current corporate system with a custom desktop application for plate processing and an OMNIS and ORACLE client/server database application, provides most of the functionality we need at the moment. Proprietary systems, however, tend to be vulnerable to loss of key personnel and to shifts in allocation of corporate resources. It is difficult to justify continuing development of functions that might be outsourced. In order to meet the data management requirements of our rapidly increasing throughput and decrease our reliance on internal computer programming resources for further software development and maintenance, we have evaluated several commercially available high throughput screening software systems This process was described.

### Accelerated structure profiling using automated LC/MS and robotics

*Jeffrey L. Whitney, Robyn A. Rourick and Kevin J. Volk, Bristol-Myers Squibb Pharmaceutical Research Institute, Princeton, NJ, USA; Saul Fink, Edward H. Kerns, Mark E. Hail and Mike S. Lee, Bristol-Myers Squibb Pharmaceutical Research Institute, New Brunswick, NJ, USA*

The pharmaceutical industry is currently undergoing initiatives aimed at accelerating the drug discovery, development and registration cycle. These initiatives have emphasized several key areas such as enhancement of productivity, early initiation of programmes, shortened time lines, parallel operations and faster go/no go decisions in an environment where prospective drug candidates and new chemical entities are expected to increase 10-fold and three-fold, respectively. Clearly, greater amounts of information produced in a fraction of the time are required to meet these demands. Analytical analyses provide critical insights throughout all segments of this cycle. Advanced analytical technologies based on standard high throughput methods are a key factor in producing this information and meeting these challenges. LC/MS is one such emerging technology that is transforming the pharmaceutical industry by providing unparalleled structural information to a wide range of analytical problems in reduced time periods.

Traditional structure analysis involves several time and resource intensive steps, such as scale-up, extraction, fractionation and detailed multi-technique spectroscopic analyses. By coupling HPLC and Tandem Mass Spectrometry, the typical bench-scale approach is transferable to a single on-line structure profiling 'engine'. In this manner one can take advantage of high sensitivity, selectivity, speed and 'universal' application capabilities inherent in these technologies. However, simply integrating these two powerful techniques is not enough. Eliminating custom method development through the use of standard methods and removing tedious manual protocols with automation are required to reduce the resource-intensive nature of LC/MS and LC/MS/MS. Additionally, automated analysis and processing methods must be combined with electronic sample tracking and results' communication to prevent information overload. With this approach over 80% of our projects can be addressed with a single set of methods. Productivity increases of 3- to 5-fold over traditional approaches have been benchmarked.

Applications of our LC/MS and LC/MS/MS-based structure profiling approach include metabolite identification, natural product elucidation, impurity/degradant identification and biomolecule characterization. LC/MS information from these analyses (e.g. retention time, molecular weight, substructure, etc.) is assembled into structure profile libraries which provide a reference for each drug candidate, linked by common standard parameters. These libraries accelerate the identification of 'in-process' drug candidates, as well as their respective impurities, degradants and metabolites. Structure libraries are also proactively produced early in the research process to predict chemical information which may provide valuable insights downstream in the process. These predictive profiles are produced by analysing samples of drug candidate exposed to various chemical environments, e.g. heat, light, acid, base, peroxide, etc.

Currently the rate-limiting steps in the predictive profile are sample production and introduction to the LC/MS instrument and interpretation of data. Initiatives are underway to automate the sample preparation, incubation and LC/MS injection steps on a Zymate XP platform equipped with several 'off-the-shelf' Zymark components. Our strategy centres around developing an investigative robotics method development station which will integrate seamlessly with our existing structure profiling methodologies. In this manner, the robotics platform will add an additional dimension, automated sample production and preparation, to our structure profiling 'engine'. Examples were shown illustrating the LC/MS-based structure profiling methods and ongoing applications of automated predictive profiling and robotics.

### Developing a flexible architecture for the Tecan Genesis liquid handling stations

*Dinesh S. Thakur, Vincent W. Chen, Robi L. Banerjee, Matthew G. Holt and Kirk J. Leister, Bristol-Myers Squibb Company, Syracuse, NY, USA*

Liquid handing stations have become an integral part of automated assays based on microplate technology. The hardware on these stations is amenable to transferring liquid in an accurate and precise manner. Integration of these stations into automated systems can be addressed in either of two ways:

- Develop analytical methods in the software supplied by the vendor of these workstations.
- Develop component software that provides complete control of the hardware through a defined interface for analytical methods development.

The first and more common approach for developing automated systems is to use the vendor supplied software. Once the analytical method is developed, the software controlling the automated system invokes this pre-programmed analytical method at run-time. This approach provides the fastest solution since the integrator does not have to deal with instrument specific details of the workstation.

The second approach is to develop component software that provides complete control of the workstation. This approach allows an integrator to program the analytical method in the software that controls the automated system. This is made possible by exposing complete functionality of the hardware of the liquid handling station through a defined interface. The upfront investment in development time provides greater flexibility for control of the liquid handling station through seamless software interfaces.

Bristol-Myers Squibb has developed a flexible architecture for the Tecan Genesis Liquid Handling Station that allows for complete control of the workstation through a defined interface. This architecture is not specific to any particular method; it is re-useable with many applications and integrates seamlessly with the software that controls the automated system. It allows for the development of systems with single interface for all peripheral hardware. This architecture is based on the principles of Object Oriented Design and utilizes the application programming interface (API) exposed by a library supplied by Tecan AG. Furthermore, this architecture addresses error handling and recovery for the Genesis workstation in greater detail than that provided by the vendor software. The specifics and the utility of the architecture implemented were discussed in this presentation.

### Automation of pH, viscosity, and colour measurements for liquid detergents

*Alp Uray and Laren Addabbo, Corporate Technology Center, Colgate-Palmolive Company, Piscataway, NJ, USA*

In the manufacture of liquid detergents, consistent and stable pH, viscosity and colour are critical to consumer acceptability. These parameters (viscosity, pH, and colour) are frequently testing during a product's development cycle as key indicators for product stability. Once established, these parameters can then be monitored to assure consistent quality when a product formula is moved into production. With the use of an articulated robotic arm, integrated bar code labelling system, custom software and customized distribution system using flow cells, a system which automates multi-sample measurement of pH, viscosity, and colour for liquid detergents has been built. This paper discussed the development of the system and results to date.

### Automated dye analyses using the Zymark BenchMate II workstation

*Rhonda J. Peischl and Marie Sabo, Clairol, Inc., Stamford, CT, USA*

Working as part of a technology service group (Analytical Research & Services) provides not only many technical challenges, but also significant challenges towards managing priorities and maintaining an adequate service level for all of our customers. Laboratory automation has played a key role for the chromatography section of the Analytical Research and Services group at Clairol by increasing productivity and increasing the number of requests that can be handled at the very same time. Simple laboratory automation such as HPLC autosamplers and programmable instrumentation have been easily introduced and utilized to conduct many unattended analyses throughout the evening hours. Automated workstations or combinations of automated instrumentation are also being linked together to aid in multi-tasking chromatographic projects. Some examples of automated workstation applications currently being employed in the chromatography labs include (1) preparing product development stability sample solutions using a Zymark BenchMate II Workstation, spotting these solutions on TLC plates using a CAMAG ATS3 (automatic TLC spotter), reading the colour of the spots using a Shimadzu CS9000U Spectrodensitometer, and then analysing the data using a computer program; (2) preparing sample solutions for safety support study(s) and transferring solutions to HPLC vials using a Zymark BenchMate II Workstation, analysing solutions by HPLC or UV/Vis; and (3) preparing processing or production troubleshooting sample solutions using a Zymark BenchMate II Workstation evaluating solutions by qualitative or quantitative TLC.

Considering the complexity and diversity of samples and requests the chromatography group continues to be challenged with on a daily basis, automation will continue to play a key role. This presentation demonstrated some of the automated workstations and applications in use in the authors' laboratory and introduced some new automation ideas.

### A Windows™ based interface for the Zymark™ robot

*Stephen Dokoupil, Helene Curtis, Inc., Rolling Meadows, IL, USA*

The development of a laboratory robotics application which connects several independent components can be a difficult job for the systems developer. Monitoring and visualization of the process activities can be simplified when efficient programming tools are used. This paper described the development of a user front end that was developed for use with the Zymark XP robot. The software operates under Windows 3.1 and was developed with LabVIEW, a programming language specifically designed for use in laboratory automation and control applications.

The software collects data from the robot as it is being generated, prints and logs the data to a data base file. The software features an intuitive graphical interface that allows manual control of the robot with mouse. When error conditions occur, the software notifies the user and allows error recovery. When testing is completed, the software will instruct the robot to put samples away and shut itself down.

This paper discussed the basic design of the software, with emphasis on the robot communications module, the user front end, and the logic flow needed for manual robot control and error recovery. The paper also discussed the advantages of programming with LabVIEW and reasons for developing in the Windows environment.

### The use of robotics in a cosmetic microbiology laboratory

*Steven F. Schnittger, Estee Lauder Companies, Research & Development, Melville, NY, USA*

The preservative challenge test is a method used to determine the efficacy of a preservation system in a cosmetic formulation. The method of testing is a labour intensive, repetitive task which consumes many hours. Product testing entails a large volume of samples which are analysed repeatedly under the same conditions and protocol. For this reason, an automated robotic system was developed to perform this testing and to free the cosmetic microbiologist to perform more meaningful and creative tasks.

Two hundred and fourteen different formulations totaling 1039 samples were evaluated comparing the automated robotic system to the manual plate count method of testing. The samples were comprised of make-ups, shampoos, conditioners, oil in water emulsions, mascaras and powders. The selection of micro-organisms tested are similar to those recommended by the CTFA Guideline for the Preservation Testing of Eye Area Products. The results showed that there was a greater than 98% correlation in results when comparing the Automated Plating System to the Manual Method of Testing. It has also been determined that the robot can save between 2–3 man hours per day.

The unattended sample preparation of the robot permits testing to continue beyond the normal workday of the job. By programming the robot to run 24 h per day, the microbiologist or technician is able to perform other functions in the lab. It also accommodates an increase in the workload of the lab, thus improving the throughput of the lab without additional personnel.

### Multi-component assembly and rapid generation of small molecule libraries on solid support

*John Perumattam, Sarvajit Chakravarty, Sandhya Suravajjala, Scios Inc., Sunnyvale, CA, USA*

A simple and general approach to the synthesis of chemical libraries based on a universal anhydride template allows the preparation of large number of compounds. Various cyclic/acyclic amines, primary/secondary amines, differentially protected bifunctional amines were used as nucleophiles to react with anhydrides. The free carboxylic acid generated was then coupled with various amines. The facile and rapid generation of this multicomponent assembly can be accomplished on solid support.

### The design, synthesis and automation of a 500 000 compound library

*M. Adam, M. Allen, S. Andrie, J. Blake, L. Charles, C. Courtney, K. Estep, S. Goldstein, M. Gross, P., Kowalczyk, J. Levine, S. Liras, J. Lorch, C. Neipp, D. Phillips, S. Robinson, E. Roskamp, D. Smith, R. Spencer, L. Stephens-Stramiello, T. Stilwill, and G. Young, Pfizer Central Research, Groton, CT, USA*

This presentation covered the library, automation design, and data management of a 500K library of druglike molecules. Library design includes desired parameters of the products, monomer selection, reaction verification and actual synthesis. Automation design encompasses equipment to handle porous tubes, error checking, software and reaction apparatus. A relational database was used to determine the path of each tube, to track the tubes and for sorting at each synthesis step. Finally, the lessons learned were discussed.

### Automated synthesis is necessary...but how to get started: fully or semi automated?

*Sigfried Brandtner and Helmut Buschmann, Grunenthal GmbH, Stolberg, Germany*

One of the essential goals of pharmaceutical chemical research is to find new active compounds. The synthesis of a screening compound requires substantial experimental effort on the part of the practicing chemist. Success in this exploratory part of research is often correlated to the number of molecules that have been synthesized, but organic chemistry is one of the most labour-intensive sciences. Combinatorial chemistry is one of the important new methologies developed by academics and researchers in the industry to reduce the time and costs associated with producing effective, marketable, and competitive new drugs. As with traditional drug design, combinatorial chemistry relies on organic synthesis methologies. The difference is the scope—instead of synthesizing large amounts of a single compound, combinatorial chemistry exploits miniaturization and automation to synthesize large libraries of compounds. The combination of high throughput screening and high throughput parallel synthesis of organic compounds has great capabilities in identifying novel drug candidates of higher quality much faster than ever before. In order to meet the increasing demands of high throughput screening for test compounds, we have been developing an automated workstation approach to have automation in place when it is necessary, not only when it is nice to have. We designed and built our automation tools the way in which organic synthesis of single compounds is carried out in the laboratory with a minimum of modification to the chemical method of reaction preparation, execution and isolation of the desired product. The design, development and realization of a multi-workstation approach was presented.

### Radio frequency encoded combinatorial chemistry—combining the advantages of parallel synthesis and the split and pool technique

*Anthony W. Czarnik, IRORI Quantum Microchemistry, La Jolla, CA, USA*

The synthesis of small molecule libraries of the order of 1000 to 10 000 members will soon be a standard job expectation in the medical chemistry laboratory. In addition to the obvious equipment requirements for miniaturization and chemical compatibility, the sheer numbers involved demand that reaction vessel handling become automated. Miniature radiofrequency memory tagged reaction vessels that are readable electronically are being developed. Work has focused on the association of tags with two different polymeric supports for synthesis: loose resin held in a rigid, porous can (MicroKan$^{TM}$) and an inert tube onto which synthesis resin is grafted (MicroTube$^{TM}$). In most instances, the tube approach appears to yield advantages in reaction agitation and washing steps, as well as in the purity of final products. The movement of reaction vessels can occur at many stages of the library synthesis process: initial reading of all tagged vessels, placement of tubes in initial reaction pots, washing, movement to subsequent reaction pots, and cleavage of products from the reaction vessels. In this presentation, the author described work using a fully manual system, as well as the development of an automated sorter capable of moving thousands of reaction vessels to individual reaction pots.

### Excel templates for reporting analytical results

*Alger Salt, Glaxo Wellcome, Research Triangle Park, NC, USA*

The author uses templates created in Microsoft Excel to report results and to process data collected from analytical instruments. Excel allows the author to perform calculations, create the reports, and export results to larger systems such as our VAX-based stability data base. Two examples, both for processing dissolution data, are MDUV and DisCor. MDUV is a software tool that improves the efficiency of transferring and processing data collected by the MultiDose workstation. DisCor applies mathematical corrections to dissolution profile data to compensate for the medium and analyte removed for off-line analysis. Both applications were described and demonstrated.

### Good development practices common to regulated and non-regulated data management systems

*J. Macdonell, Taratec Development Corporation, Bridgewater, NJ, USA*

Standards and procedures developed to insure the integrity of systems and data utilized in the regulated environments offer value and controls that can be extended to non-regulated environments.

Various GxP regulations require established methodologies and practices in the development, implementation, and operation of regulated systems. It is acknowledged that these agreed upon methodologies and practices also achieve business value in terms of reduced risk, controlled development, advanced user knowledge of the systems capabilities, and forecasted cost control and return on investment.

This presentation discussed how guidelines established to meet FDA requirements can be effectively and efficiently applied to the development of other systems. These guidelines provide a known starting point and baseline under which all systems can be implemented. Should users need to utilize such systems in a regulated environment the known steps to a validated system are clear and achievable. Further, the established controls lead to better and more user focused systems. The lessons learned regarding what works well and what areas are of greatest concern were explained.

### Data management industry task force summary

*Matt Citardi, Barr Laboratories, Pomona, NY, USA*

An Industry Task Force on Automation and Data Management was formed in April 1997. The group consisted of representatives from the pharmaceutical industry and was facilitated by Zymark's PDF Team. The group met to discuss current issues relating to laboratory automation and data management. Use of automated sample preparation instruments is known to create a new bottleneck in laboratory workflow. Automation and robotics can alleviate the backlog of samples created by sample preparation and analysis requirement. In the majority of situations, manual labour is required to review and transfer the large amount of data generated into a results reporting program. Through formal interviews and interactive discussion the common constraints and problems with this approach were identified. The Task Force developed potential solutions to the problems, focusing on technologically feasible solutions that fit within the corporate data strategies. The Task Force findings were presented and followed by a panel discussion.

### Ultra-high-throughput screening with 1536 wells! Where is the physical limit in well size?

*G. Knebel, Greiner Labortechnik GmbH, Frickenhausen, Germany*

The increasing demand for miniaturized assays for high-throughput screening has been the driving force in designing 384-well plates with the same footprint as a 96-well plate. The 384-well plate incorporates a quadruple higher well density with a one-half well to well spacing of 4.5 mm. The decision to make square instead of round wells has reduced total volume to a third (120 µl); routine working volume is 30–90 µl.

The skill and efforts of technical staff has resulted in further miniaturization. Greiner has developed a tool technology to produce a 1536-well plate (four-fold well density of a 384-well plate) in white opaque and black polystyrene with injection molded quality. The total volume of the square wells is approximately 13 µl, the well spacing is 2.25 mm.

Even with sophisticated techniques for high precision molds, the latest software for injection molding machines, and resins with excellent flow properties, it is merely impossible to manufacture ultra-high density plates in the µl-range with more than 6144 wells in a technically traditional way.

This presentation focused on several novel microplates ranging from 96 up to 6144 wells which have been recently developed to cover existing and future demands. These include thin-walled clear bottom plates with excellent optical properties and quenched crosstalk, UV-transparent plates with a superior transmission window down to 250 nm and high chemical resistance.

### Automated solutions for achieving the goals of high-throughput screening at Wyeth-Ayerst Research

*James La Rocque, Wyeth-Ayerst Research, Pearl River, NY, USA*

A commitment of resources to HTS at Wyeth-Ayerst has resulted in an expansion of laboratory space and personnel, as well as the acquisition of efficient automation necessary to achieve the goal of screening a 200 000 sampling library in several different assay formats. The implementation of a variety of screens on Zymark integrated systems, as well as workstation-based approaches to screening were reviewed. The generation of daughter sample plates on a dedicated Zymark system were also reviewed.

### A multipurpose dual assay robot for compound handing and drug discovery

*Brent T. Butler, Joan Frezza, and Julie J. Tomlinson, GlaxoWellcome Inc., Research Triangle Park, NC, USA*

The Zymark scheduling software PCS allows the robot operator to program true multitasking into the system. The ability to do more than one job a time (i.e. compound handling and biochemical assays) provides increased throughput. The system can perform both compound handling and biochemical assays in parallel. This paper covered onsite integration and implementation of the multifunctional system, as well as some applications which have been employed by the system.

### High-throughput, mechanism-based screening techniques for discovering novel agrochemicals

*James C. Walsh, American Cyanamid Company, Princeton, NJ, USA*

A variety of screening strategies can be employed by discovering novel agrochemicals such as fungicides, insecticides, herbicides and antiparasites. Traditionally, random evaluation of chemical and natural products samples has been used in assay systems ranging from greenhouse testing down to *in vitro* microplate screening. This task can be formidable depending upon the size of sample libraries and personnel resources available. One important tool in the overall discovery process at Cyanamid is the application of highly specific and sensitive mechanism-based assays for the purpose of identifying novel leads.

This paper described high throughput, agar-based screening techniques successfully implemented in a decentralized screening environment where laboratory space and staffing are limited. Through the prudent use of automated and manual high density agar-based techniques, multiple sources of samples can now be processed and evaluated against multiple targets in a timely fashion. Assay validation can be streamlined. The advantages and disadvantages of an agar-based screening approach were discussed.

### Robotic method for semi-automation of plasma triglyceride structure analysis by lipase

*R. O. Adlof, L. C. Copes and E. A. Emken, USDA, ARS, NCAUR, Peoria, IL, USA*

Our studies on the absorption and metabolism of triglyceride (TG) positional isomers in human and animal models required the analysis of isolated triglyceride samples by lipase. The triglycerides of blood and isolated blood fractions, mouse liver tissue, native/randomized lard and soybean oil were extracted and treated with lipase. The 2-monoglyceride (MG) fractions were separated from TGs and diglycerides (DGs) on a Zymark BenchMate Workstation by silica SPE (3 ml Bond Elute© columns). TLC was used to verify the completeness of MG separation. Average recoveries (*c.* 40%) of the 2-monoglycerides in $>95$ purity were consistent with published results. The isolated MGs were manually converted to methyl esters. The workstation (hexane as extracting solvent) was used to isolate the methyl ester fractions from the reaction mixtures. The impact(s) of solvent volumes, mixing times and sample sizes on MG and ester recoveries was studied. Gas chromatography was used to determine fatty acid compositions; percent recoveries were calculated by the addition of known weights of 17:0 and 21:0 internal standards to the samples. Ester recoveries for the BenchMate Workstation were dependent on the initial substrates and varied from 65% to $>90\%$ compared to manual extraction data. Fatty acid compositions of ester samples isolated by robotic methods agreed with 0.5% with samples isolated by non-robotic means. Oxidation products were not a problem. Solvent removal from the isolated samples was done with a Zymark TurboVap LV Evaporator Workstation.

### The utilization of multiple component analysis by LC-MS/MS in *in vivo* pharmacokinetic screening experiments early in the drug discovery process

*Timothy V. Olah, Merck & Company, West Point, PA, USA*

LC-MS/MS has been rapidly embraced by the pharmaceutical industry as the definitive method for the determination of drug levels in biological fluids obtained from pharmacokinetic and toxicological studies. This technique has proven to be reliable, accurate and precise for the determination of drugs and related substances (metabolites and isotopes) in support of preclinical and clinical studies. Our group has recently expanded the use of quantitative LC-MS/MS into the area of discovering new substances as potential drug candidates. It is well documented that when used as an accurate mass detector, triple quadropole instruments have the ability to simultaneously and specifically detect minute quantities of closely related drug substances in the extracts of biological fluids. As the development of synthetic techniques advances, we have focused our attention on the development and implementation of analytical methods that can simultaneously measure plasma concentrations of up to 22 drug candidates over a concentration range of 1–1000 ng $ml^{-1}$ in single analytical occasions. The accuracy and precision of easy assay is determined following the evaluation of results obtained from quality control samples which are incorporated in each analysis.

This approach is used to support drug discovery by rapidly providing pharmacokinetic data to a wide range of compounds by either increasing the speed and efficiency of analysing samples following the administration of single components to multiple animals or, by the rapid assessment of plasma levels determined for each individual component following the administration of multiple compounds to single animals. These co-administration studies are carried out using compounds that are either synthesized individually and combined for dosing to animals or, synthesized as intact combinatorial libraries consisting of up to 20 compounds.

Currently, there is great interest in establishing technology for automated high-throughput bioanalysis involving both pharmacokinetics and metabolic stability within the pharmaceutical industry. There are several advantages and disadvantages to incorporating multiple component analysis into a bioanalytical laboratory. These include the rapid generation of high quality analytical data and the efficient use of animals and instrument time, as well as the limitations of assay sensitivity, the speed of data processing and time, as well as the limitations of assay sensitivity, the speed of data processing and reporting and the potential for drug interactions complicating the interpretation of results.

All work presented was performed under Good Laboratory Practice (GLP) guidelines.

### Precise bioanalysis using the RapidTrace workstation: application to an HPLC-UV method for a new antihistamine and an LC-MS/MS method for polypeptide

*Roger A. Coe, Hong Zhang, Mary E. Petersen, Jeff D. Moran, Patrick Lin, and Jean W. Lee, MDS Harris, Lincoln, NE, USA*

An automated SPE RapidTrace Workstation has been used at MDS Harris for routine bioanalytical sample analysis. Extraction of different types of analytes from biological fluids using a variety of SPE columns has been developed. Illustrations were given for two methods: a new antihistamine extracted with C18 cartridge, isolated and quantified by a heart-cut column-switching, ion-pair, reversed-phased HPLC-UV method; and A polypeptide extracted with Water Oasis™ cartridge and quantified by a Perkin Elmer AP1300 LC-MS/MS in the negative ion mode. The methods were validated and applied to clinical sample analysis. The methods were shown to be precise, accurate, rugged and highly productive.

### Robotics and information systems integration

*Jean Mercier, The Surrey Research Park, Guildford, UK*

The integration of the screening chain from preparation, selection, assay run, acquisition, analysis, storage and retrieval of information plays an important role in the increase of the drug discovery productivity (through the amount of accurate hits generated) in screening laboratories.

Data management software is not just a tool for the management of compound related data (chemical and biological). It is the central component of an information management system used to facilitate research and R&D decision-making, making it a decision enabling tool. Being such an important and central tool in pharmaceutical R&D, the quality of the data in data management systems needs to be of the highest standards. The quality of data is directly related to data integrity. However, the control software in today's automated platforms have not been designed to sufficiently guarantee data integrity. Sometimes no control is available at all or control is maintained through sets of data files with one file containing the linking information. Some systems provide interpreted data (calculated results) only, with limited possibilities to control and track raw data. IDBS and Zymark have developed a standard interfacing scheme to ensure the quality of data, data integrity and control and tracking of raw data. The integration between information system software and workflow controller software have multiple applications. Control of data flow, increase of data accuracy during process, increase of screening throughput automation during the confirmation phase.

### Modular workstations for automated solution phase synthesis

*M. Brands, M. Es-Sayed, V. Grundel, P. Naab, T. Repp, U. Rosentreter, C. Schmeck, R. Schmitz, W. Schrock, Bayer Pharma-Research, Wuppertal, Germany*

Workstations for parallel synthesis in solution and sequential purification of precursors, templates and final compounds were described. Automated processes are worked off interactively with the user to maintain a high degree of flexibility. Therefore, these workstations can be used for methodology, preparation and purification of larger and minor quantities.

Consequently these workstations are used, when small to medium sized libraries are needed for immediate hit evaluation and directed synthesis for lead structure optimization.

Synthesis is accomplished on a liquid handler based workstation, which is also used for methodology. This workstation can handle air sensitive materials and covers a temperature range from −20 to 150°C. A number of different reactions have been applied to the machine, of which some were presented.

Depending on the scale the crude material is purified by automated flash chromatography (20 mg–10 g) or automated HPLC (5-20 mg.) For flash chromatography 11 samples can be purified sequentially in one run. Again a liquid handler is used for fraction collection and sample preparation for analysis or biological testing. By HPLC up to 96 samples are separated in one run.

Partially automated processes simplify software and hardware design dramatically and thereby handling and maintenance, especially when interchangeable parts are used. Special training is thereby reduced to a minimum. The ultimate goal is to use such workstations everywhere by everyone in chemistry.

